# Subtyping depression using brain–gastric electrophysiology for early prediction of antidepressant response: a multicentric clinical study

**DOI:** 10.3389/fpsyt.2026.1823806

**Published:** 2026-07-03

**Authors:** Amal Jude Ashwin Francis, Alok Bajpai, Nandini Priyanka Balasubramani, Hari Prakash Tiwari, Shikha Singh, Kritika Chawla, Ayushi Devendra Singh, Dhananjay Chaudhari, Pragathi Priyadharsini Balasubramani

**Affiliations:** 1 Department of Cognitive Science, Translational Neuroscience and Technology Lab, Indian Institute of Technology Kanpur, Kanpur, India; 2 Department of Psychiatry, Ganesh Shankar Vidyarthi Memorial (GSVM) Medical College, Kanpur, India

**Keywords:** biotypes, depression, early prediction, electroencephalography, electrogastrography, gut-brain coupling, longitudinal assessments, treatment outcome

## Abstract

Depression affects approximately 5% of adults worldwide, with India reporting a prevalence of 4.5%. Oral medication is a common treatment, but over 50% of patients fail to respond to first-line antidepressants and often require medication adjustments or augmentation. This highlights the urgent need for predictive models that can guide personalized treatment strategies more quickly. Our study aims to achieve three key objectives: first, to assess the predictive ability of previously identified biomarkers such as frontal theta power and alpha asymmetry in explaining the response to interventions within our sample; second, to evaluate the utility of a holistic research approach focusing on gut–brain interactins, in predicting responses; and third, to identify reliable early biomarkers that can predict responses across various phenotypic subtypes characterized by dominant clinical presentations. Across two sites (site 1 + site 2), a total of 206 (161 + 45) participants, including 144 (99 + 45) treatment-naive patients, were enrolled in our study, which spanned three visits. We aimed to predict antidepressant outcomes at the third visit (4–6 weeks) using data collected from visits one (baseline) and two (7–10 days), and the data from site 2 were solely used for testing the predictive utility. Our predictive models, which incorporated electrophysiological data from both the brain and gut along with clinical information, achieved cross-validation (holdout test) performance of 78% (80%) specificity and 84% (71.43%) sensitivity in identifying nonresponders to antidepressant treatment administered as per Clinical Practice Guidelines of India. We found that certain electrophysiological features were strongly predictive of treatment outcomes for specific depression subtypes. For example, increased excitation–inhibition ratios in the frontocentral brain regions were predictive of patients with dominant anxiety and sleep symptoms. Similarly, decreased tachygastric coupling with the sensorimotor brain region predicted treatment nonresponse in patients with high levels of negative self-thoughts. Increased connectivity in the right frontocentral region was associated with better outcomes in patients with significant appetite issues. Additionally, higher frontocentral theta power and beta asymmetry were predictive of responses in patients with a composite set of symptoms. Our findings suggest that combining brain and gut electrophysiological markers with clinical phenotyping offers a promising, scalable approach to personalize depression treatment. This approach could guide clinicians in developing more effective and tailored medication strategies, ultimately improving patient outcomes.

**Clinical trial registration:**

Clinical identifier Trial Registry of India https://ctri.nic.in/Clinicaltrials, CTRI/2025/04/084404.

## 1 Introduction

According to the World Health Organization (WHO), Major Depressive Disorder (MDD), commonly known as depression, is a mental health condition characterized by persistent feelings of sadness, lack of motivation, and an inability to experience pleasure. Key symptoms of depression include disrupted sleep and appetite, difficulty concentrating, feelings of hopelessness, low energy, and thoughts of suicide. The current standard treatment for depression involves selective serotonin and norepinephrine reuptake inhibitors (SSRIs/SNRIs). However, this approach typically follows a trial-and-error process in which patients are prescribed medications, but it can take 4 to 6 weeks to assess their effectiveness. Unfortunately, nonresponse or nonremission rates are high—around 50%–60%—following the initial treatment trial. Many patients either experience side effects or find that the medication does not effectively alleviate their symptoms, which leads to further adjustments or changes in the treatment regimen (1–4).

There is a notable gap in the availability of neurophysiological tools that can accurately predict treatment outcomes within a short timeframe—less than 2 weeks—while accounting for the neuroplastic effects of depression. Earlier attempts utilized a combination of clinical health records, behavioral data, and genomic information to achieve a commendable efficiency rate of around 72% within a 4-week timeframe. Foundational frameworks have further emphasized the potential of neurophysiological markers in guiding treatment selection (5). Large-scale studies such as Sequenced Treatment Alternatives to Relieve Depression (STAR*D) highlight variability in treatment outcomes, underscoring the need for an improved predictive model (6–9). Relatedly, there are a few studies that look into neurophysiology at any time point to predict depression severity (10–12), and claim to achieve greater than 90% accuracy.

Large randomized clinical trials, such as EMBARC, have shown that higher baseline theta-band activity in the anterior cingulate cortex and its connectivity with salience-related brain networks are associated with better responses to sertraline treatment (13, 14). Data from the STAR*D study further emphasize the importance of early response as a predictor of remission with citalopram treatment (15). Several studies, nearly a decade old, tracked changes in neural activation due to medication and found that increased frontal theta power and reduced frontal alpha asymmetry were key markers of treatment response, even for medication-resistant patients, achieving over 91% accuracy when combined with depression rating scale features (16). Moreover, frontal beta power has also been linked to depression (17, 18).

However, more recent studies over the past 5 years have presented contradictory findings. Some research suggests that frontal alpha asymmetry is not sensitive to treatment response (19–21), while other studies argue that it is not low-frequency power increases, but rather aperiodic activity, that better explains treatment responses in patients (22). These conflicting views raise concerns about the reliability and robustness of previously identified neurophysiological markers. Several clinicians have called for a reform in depression treatment strategies, incorporating biological insights, as outlined in the Research Domain Criteria (23). A more comprehensive phenotypic profiling of neural circuits and physiological mechanisms, with precision intervention targets, may significantly enhance remission rates in depression treatment.

In recent years, growing evidence suggests that depression severity is not solely reflected in brain physiology but also in whole-body physiology. Gut dysfunctions, such as irritable bowel syndrome and gastroparesis, often confound depression symptoms (24–27). This has led to an increasing recognition of the gut-brain interaction as crucial to understanding depression symptoms (28–30). Despite this, gut-related markers have not been incorporated into depression research or clinical treatment strategies.

Our study introduces a novel approach by integrating gut–brain cognition, including gut–brain coupling and electroencephalography (EEG), to predict treatment response. Additionally, we incorporate cognitive task probes to better understand how neural circuits are recruited during various tasks, which can aid in predicting treatment outcomes. Specifically, we hypothesize that combining physiological data from the brain and gut during cognitive tasks, along with clinical and demographic information, can enable early prediction of treatment outcomes in depression, well before the typical 4–6-week assessment period. A key aspect of our work is the identification of phenotypic subtypes within depression, which has allowed us to uncover distinct neurophysiological markers linked to specific symptom clusters. We found that different subtypes of depression, defined by symptom dominance (e.g., anxiety, sleep issues, negative self-thoughts, or appetite changes), show brain and gut signatures that predict treatment response.

By combining phenotypic profiling with electrophysiological biomarkers, our predictive model can offer personalized treatment guidance—identifying early responders and nonresponders as early as 7–10 days into treatment. This contrasts with the traditional 4–6-week evaluation period and could significantly enhance clinicians’ ability to tailor interventions more effectively and quickly. Ultimately, our study offers a scalable, precision-based framework that integrates both brain and gut data, as well as clinical phenotyping, to improve treatment outcomes in depression.

## 2 Methodology

**Figure 1:**
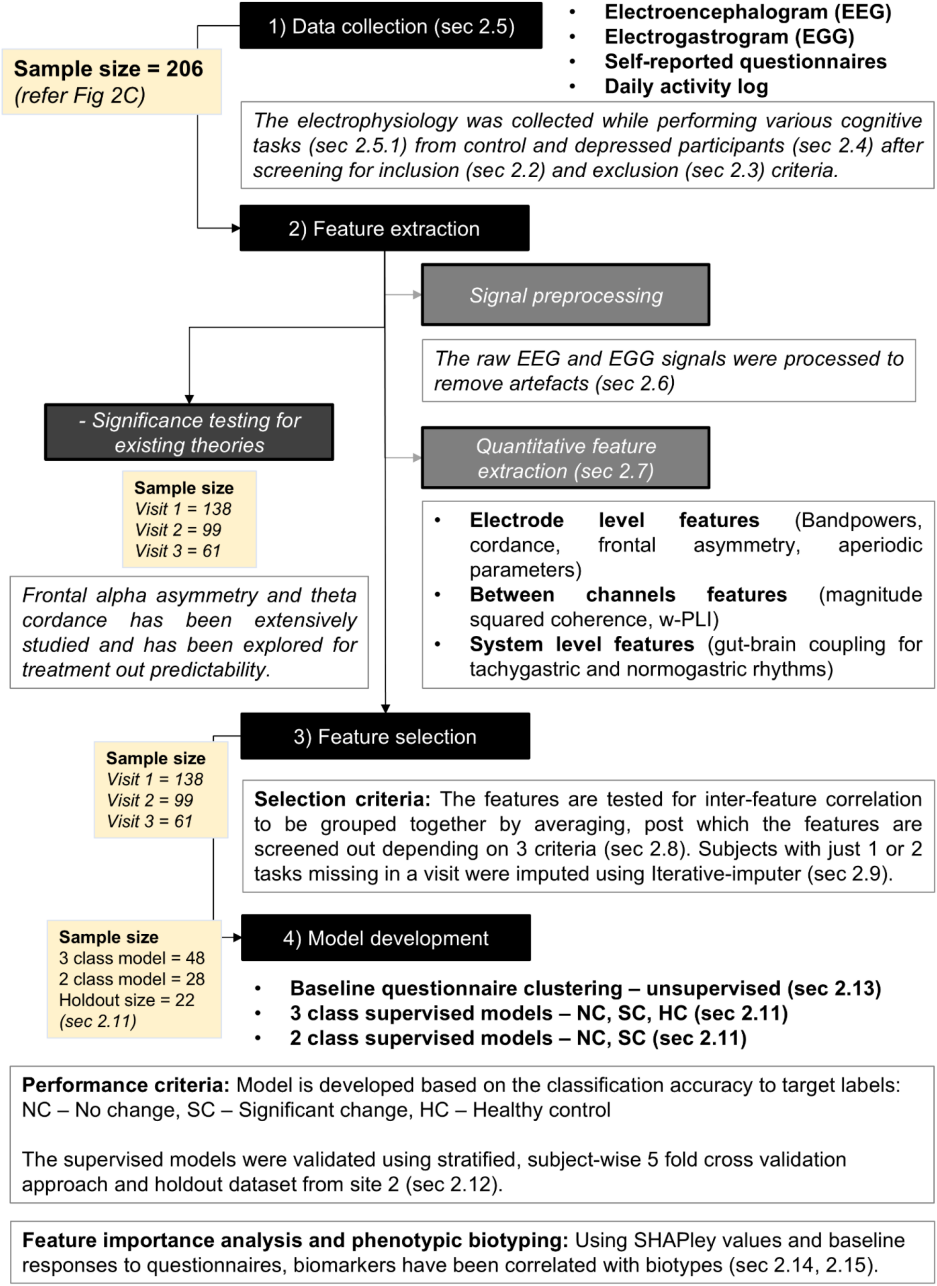
Overview of methodological workflow. The table summarizes the major methodological components of the study, the number of unique participants contributing data to each module, and a brief description of their methodological relevance.

This section can be broadly divided into four subsections, namely (1) data collection, (2) feature extraction, (3) feature selection, and (4) model development. In the data collection, the experimental setup and the tools used for collecting longitudinal electrophysiological data, as well as self-reported questionnaires, are described. In the feature extraction, the quantitative features derived from the collected electrophysiological data are discussed elaborately. In the feature selection, the methods used to identify and select relevant features for model development and analysis are outlined. In the model development, the machine learning models used for classifying clinical participants into responders/nonresponders are presented. A detailed methodological overview, including the sample size used for each step, is provided in **Figure 1** for reference.

### 2.1 Data sites, ethical clearance

The clinical study data were acquired from two independent sites. The model prototype was developed using data acquired at the Indian Institute of Technology, Kanpur site (site 1), in collaboration with the institute psychiatrist, who sees patients via his psychiatry clinic. Ethical clearance for the study was obtained from the institutional ethics committee of the Indian Institute of Technology Kanpur in February 2022.

Based on the above study, a clinical trial was planned at an independent site, Ganesh Shankar Vidyarthi Memorial (GSVM) Medical College, Kanpur (site 2), and registered in the Clinical Trial Registry of India (CTRI/2025/04/084404). Ethical approval from the GSVM ethics committee was obtained under the registration number EC/BMHR/2025/28. The data acquired from this site were used solely for an entirely held-out evaluation of the previously developed predictive model presented in this manuscript.

Informed consent from the participants was obtained both orally and in written form. The participants were awarded coupons for each visit and were allowed to voluntarily withdraw from the study at any time without any deduction in compensation for the visit. Data confidentiality was maintained, and a subject ID was assigned to participants to ensure anonymity prior to further processing of the data.

### 2.2 Inclusion criteria

Healthy young adults from the university campus and community settings, such as schools and colleges, were included as a control group. The control population refers to individuals not seeking any clinical help and not diagnosed with any mental disorders. Treatment-naive patients were recruited for the depression group. Participants of all handedness and genders were included. Depressed participants refer to those clinically diagnosed according to ICD-10 DCR with a HAM-D score ≥ 14 and who received unimodal medication treatment during the time of study. The age range of 18–60 was a criterion for inclusion.

### 2.3 Exclusion criteria

Patients who were in their pregnancy or postpartum period, with any neurological or medical comorbidity that could influence research investigations, were excluded from the study. Additionally, for depressed patients, participants with suicidal intent or any psychiatric emergency, bipolar depression, psychotic symptoms, or substance use disorder were excluded from the study. Participants with a multimodal treatment strategy were excluded from the study.

### 2.4 Participant consort

**Figure 2:**
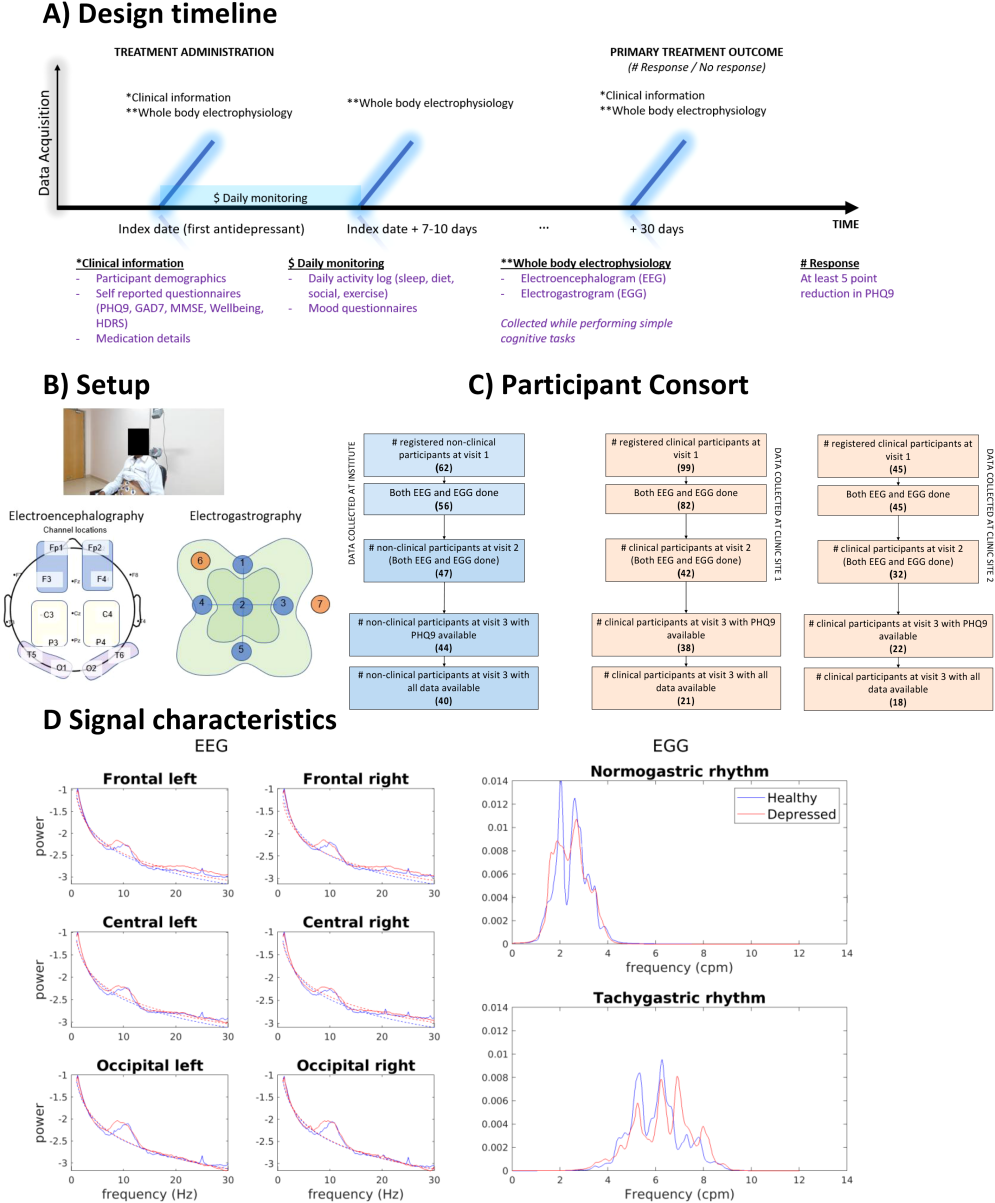
Study design and baseline electrophysiology characteristics. **(A)** Study timeline: In visit 1, clinical information such as demographics, questionnaires, and baseline brain–gut electrophysiology (electroencephalography [EEG], electrogastrography [EGG]) data are collected. During visit 2, only brain–gut electrophysiology while performing simple cognitive tasks is collected. In the final visit, self-reported questionnaires and brain–gut physiology are collected. **(B)** Experimental setup: The grouping of EEG electrodes in six regions and the location of electrodes for the electrogastrogram setup are depicted. **(C)** Participant consort: The number of participants for each visit with electrophysiology and PHQ-9 questionnaire data is highlighted. **(D)** Averaged power spectral density curves for clean EEG and EGG across tasks: Log-transformed PSD and aperiodic fit (dotted lines) for the filtered band range of healthy and depressed populations are plotted for comparison. Central theta EEG was significantly greater in the depressed population. In EGG, we present the normogastric (0.03–0.07 Hz) and tachygastric (0.07–0.15 Hz) power for the two groups, and the normogastric signals were higher in the control group.

From site 1, out of 161 participants, electrophysiological data from the brain (electroencephalogram [EEG]) and gut (electrogastrogram [EGG]) were collected from 138 participants, including both control and patient populations at the baseline visit. However, only 62 participants completed all three visits, and EEG and EGG were collected from them. Baseline visit (visit 1) was conducted on day 0, intermediate follow-up visit (visit 2) occurred between 7 and 14 days thereafter, and final follow-up visit (visit 3) was conducted after 30–40 days (refer to **Figure 2A**).

Patient population refers to participants who are clinically diagnosed with depression, are naïve to medication intake, and are receiving clinical care specifically through oral medication (**Figure 2C**). In cases of prior antidepressant history, careful medication strategies, such as tapering with a 1-month washout period, were used to account for the individual medication history of the patients. Medication was chosen according to the judgment of the attending physician and in accordance with the Clinical Practice Guidelines (CPG; 31). All participants had normal or corrected vision. No other treatments were prescribed to the patient or control populations. A small token of gratitude was provided to the control group for their participation. As the study was conducted in a real-world setting, the choice of medications was not controlled by the investigators.

From site 1, 62 healthy and 99 clinical participants registered for the study. Among the healthy participants, 47 completed visit 2, 44 completed the self-reports at visit 3, and only 40 provided physiological data at visit 3. Among the clinical participants, 42 completed visit 2, 38 completed the self-reports at visit 3, and only 21 provided physiological data (EEG, EGG) at visit 3. From site 2, 45 patients registered in total by October 2025, 32 completed visit 2, 22 completed the self-reports at visit 3, of whom only 18 provided physiological data at visit 3 (please refer to **Figure 2C** and the workflow diagram [**Figure 1]** for more details).

For statistical testing of existing theories and their ability to predict treatment response, data from 138 participants at site 1 were used at visit 1, 99 at visit 2, and 61 at visit 3. For machine learning model development, participant inclusion was determined based on the classifier target label definitions (**Table 1**). A total of 48 participants from site 1 met the criteria and were included in the three-class model development, while a subset of 28 participants (comprising only significant change [SC] and no change [NC] groups) was used for the two-class models. For complete hold-out validation, 18 participants from site 2 who completed all three visits were included. In addition, four participants with valid electrophysiological data up to visit 2 and available Patient Health Questionnaire-9 (PHQ-9) scores at visit 3 were also incorporated, resulting in a holdout sample of 22 participants with both valid input features and ground-truth labels.

**Table 1:** Terminologies used to define the participants based on their symptomological profile.

Site of recruitment	PHQ-9 ≤ 5 (visit 1)	PHQ-9 > 5 (visit 3), ≤ 30% reduction (visits 1–3)	PHQ-9 > 5 (visit 3), < 30% reduction (visits 1–3)
Nonclinic (control)	Psychotypical healthy controls (HC)	Nonclinical responders	Symptomatic controls (NC)
Clinic (patient)	Subthreshold depression	Significant change in mental health (SC)	No change in mental health (NC)

The groups are categorized as HC/SC/NC and are used for model development and analysis. The rows indicate distinct sites of participant recruitment, while the first column indicates the status of initial visit 1 PHQ9, and the second and third columns indicate the final visit 3 PHQ9 scores.

### 2.5 Data acquisition

Electrophysiological recordings, responses to questionnaires, daily activity logs, prescription information, and demographics were collected across three visits: day 0 (baseline visit), days 7–14 (visit 2), and weeks 4–6 (visit 3), as needed and described below.

#### 2.5.1 Visit-wise workflow overview

During the first visit, after obtaining written consent, the participant answers the appropriate self-reported questionnaires (PHQ-9, General Anxiety Disorder-7 [GAD-7], Mini-Mental State Examination [MMSE], Hamilton Depression Rating Scale (HDRS), well-being), followed by electrophysiological data acquisition through EEG and EGG. Data were recorded and saved while participants performed resting-state, breathing, and photic tasks, as described below. During the second visit, participants were asked to perform the same tasks as in visit 1, along with working memory and MMSE assessments, while electrophysiological data (EEG, EGG) were simultaneously collected. A similar procedure was followed for visit 3 in terms of electrophysiological data recording, and the clinical questionnaires were administered again, as in visit 1 (PHQ-9, GAD-7, HDRS; refer to **Figure 2A**).

#### 2.5.2 Task setup

The participants performed simple tasks that have been reported to evoke various cognitive processes, such as interoception, attention, memory, arithmetic calculation, and language.

##### 2.5.2.1 Resting-state tasks

Resting-state, eyes-open, and eyes-closed tasks were performed for approximately 5 min each while participants were resting on a chair in a quiet setting.

##### 2.5.2.2 Breathing task

An interoceptive breathing task was then performed, in which participants were asked to focus on their breathing, hyperventilate, and take deep breaths while holding their breath for approximately 5 s, repeated about 10 times over a total duration of 2–3 min.

##### 2.5.2.3 Photic administration task

A varying-frequency photic task was administered, in which the photic stimulus, presented at the upper-right visual field of the participant, flickered at 3, 5, 7, 9, 11, 13, 15, 17, 19, and 21 Hz, with each frequency presented for 10 s, an interfrequency interval of 1 s, and a total duration of 110 s.

The abovementioned four tasks were performed across all three visits. During visits 2 and 3, in addition to the four tasks, participants performed a picture description task and an arithmetic task involving counting backward in multiples of 7 from 100 to 40. In the present study, only the four spanning all three visits were used for analysis. Mini-Mental State Examination was administered, and the results were stored to assess different cognitive abilities of the participants.

#### 2.5.3 Self-reported and expert-administered questionnaire data acquisition

We administered self-report questionnaires such as PHQ-9 (32), GAD-7 (33), MMSE (34), and expert-administered HDRS (35) during visits 1 and 3, as well as the well-being scale (36), along with the collection of electrophysiological measures. A daily activity log was administered between visits 1 and 2, and its data were not used in the present study.

#### 2.5.4 Electrophysiological data acquisition

Electrophysiological signals were obtained while participants performed all simple cognitive tasks while resting comfortably in a chair. EEG was recorded using a 24-channel montage comprising 22 recording electrodes, one reference electrode, and one ground electrode, following the 10–20 system, using the BrainTech system manufactured by Clarity Medicals Pvt. Ltd. Punjab, India. The data were sampled at 256 Hz. EGG was recorded using an OpenBCI setup with one ground, one reference, and two recording electrodes, placed as mentioned in the figure (**Figure 2B**). The data were acquired at a sampling rate of 250 Hz. The same setup was used across all three visits. For about 23 participants who were recruited during the final stages of the study, a seven-channel EGG setup was used, while a five-channel setup was used for the remaining participants.

### 2.6 Electrophysiological data preprocessing

#### 2.6.1 EEG

EEG data cleaning was performed using EEGLAB v2022.1, MATLAB R2022b.

##### 2.6.1.1 Bandpass filtering

The EEG data were bandpass filtered using a Hamming windowed FIR filter (pop_eegfiltnew function in EEGLAB), with the “locutoff”, “highcutoff”, and “filtorder” parameters set to 0.5, 35, and 3, 300, respectively. Slow-frequency drifts and high-frequency channel noise were removed during bandpass filtering.

##### 2.6.1.2 Labeling bad channels

Bad channels were labeled postfiltering based on whether the standard deviation of any channel exceeded the 75th percentile of the standard deviation of the remaining channels and was greater than 100 or less than 1 µV.

##### 2.6.1.3 Source localization-based artifact removal

Independent component analysis (ICA) was performed on the data after removing bad channels using the pop_runica function with “icatype” parameter set to runica. For *n* channels, ICA yields at most *n* components. Component labels were obtained using the iclabel function in EEGLAB, which classifies components into “brain”, “eye”, “muscle”, “heart”, “line noise”, “channel noise”, “others”, and returns the predictive probabilities for each class. Bad components—defined as those with a sum of predictive probabilities for brain and others less than 0.1—were removed from the channel signals using the pop_subcomp function in EEGLAB.

##### 2.6.1.4 Interpolation of bad channels

The labeled bad channels were interpolated using the pop_interp function, with the “method” parameter set to spherical.

##### 2.6.1.5 Common average referencing

Finally, common average re-referencing was performed using the pop_reref function. Common noise recorded across all channels was reduced using the common average referencing method.

##### 2.6.1.6 *Z*-score normalization

The resulting EEG signals for each task and subject were *Z*-score normalized across all channels.

#### 2.6.2 EGG

##### 2.6.2.1 Bandpass filtering

To obtain a normogastric signal, the EGG signal was bandpass filtered between 0.03 (1.8 cycles per minute [cpm]) and 0.07 (4.2 cpm) using the fir2 function in MATLAB, with a transition width of 0.01 and a filter order of 3. For tachygastric signals, the lower and upper frequencies were set to 0.07 (4.2 cpm) and 0.15 (9 cpm), respectively (37). The power spectral densities of the filtered signals are used to validate the filtering process (**Figure 2D**).

### 2.7 Quantitative electrophysiological feature extraction

The data were fragmented into 1-min fragments, and quantitative features were extracted from the preprocessed EEG and EGG. The EEG features were grouped region-wise into six regions: frontal right (Fp2, F4), frontal left (Fp1, F3), central right (C4, P4), central left (C3, P3), occipital right (O2, T6), and occipital left (O1, T5).

#### 2.7.1 Absolute band power of brain signals

The power spectral density was computed from the *Z*-score-normalized channel signals using a fast Fourier transform with a 5-s window size and 50% overlap. The average band power (theta: 4–7 Hz, alpha: 8–12 Hz, and beta: 13–30 Hz) was calculated as absolute band power within each frequency range.

#### 2.7.2 Relative band power of brain signals

The relative band power was computed by dividing the absolute band power by the total power between 1 and 35 Hz, as this range encompasses all considered frequency bands and falls within the bandpass filtering range.

#### 2.7.3 Separation of aperiodic and periodic band power in brain signals

The power spectral density (PSD) was obtained using the pwelch function in MATLAB, with 5-s windows and 50% overlap between windows, and was normalized using Equation 1. Brain signals recorded using EEG exhibit a 1/f component, i.e., higher power at lower frequencies and lower power at higher frequencies. Therefore, the fitting oscillations one over frequency (FOOOF) method was used to separate the aperiodic and periodic components of the power spectral density (38). FOOOF fits two aperiodic parameters, the exponent and offset, to the log-transformed power spectral density of the broadband EEG signal and generates an aperiodic component. After removing the aperiodic component, the periodic component is obtained and is comparable across frequencies. Band powers were computed using only the periodic component. The aperiodic parameters, offset and exponent, were also stored after fitting oscillations one-over frequency.

(1) $${\text{Power}}_{f_{\text{norm}}}\;=\;\frac{{\text{Power}}_{f}}{\sum_{i\;=\;0.5}^{35}{\text{Power}}_{i}}$$

#### 2.7.4 Approximate entropy

Approximate entropy is a measure of randomness in a signal. It was calculated using the approximateEntropy function in MATLAB, with the lag set to 1 s. Higher values of approximate entropy indicate greater randomness, whereas lower values indicate higher predictability and reduced randomness.

#### 2.7.5 Theta cordance

Historically, theta cordance has been used as a measure of energy consumption in a given region (39). It is calculated as the sum of absolute theta power and relative theta power, as computed in Sections 2.8.1 and 2.8.2, respectively.

#### 2.7.6 Band power asymmetry

Band power asymmetry between the right and left regions is defined as the difference in their relative band powers. A negative value indicates greater band power in the left region, whereas a positive value indicates greater band power in the right region.

#### 2.7.7 Magnitude-squared coherence

Magnitude-squared coherence computes the cross-power spectral density between two signals and provides insight into the similarity between their PSDs. If a particular frequency is present in both signals A and B, the coherence at that frequency is closer to 1; similarly, if it is absent in both signals, it is closer to 1. In any other case, the coherence value is low and tends toward 0. In this study, coherence was computed using the mscohere function in MATLAB using clean broadband EEG signals from the electrodes. Coherence within specific bands was computed by averaging coherence across the frequencies of interest (Equation 2).

(2) $${\mathrm{Coh}}_{AB}\left(f\right)\;=\;\frac{|P_{AB}\left(f\right){|}^{2}}{P_{AA}\left(f\right)*P_{BB}\left(f\right)}$$

Where the numerator term is the cross-power spectral density between signals A and B at frequency “f”, and the denominator is the normalizing term used to limit the coherence value between 0 and 1.

#### 2.7.8 Weighted phase lag index

Weighted phase lag index (PLI) between two signals is a measure of the presence of a consistent phase difference after accounting, to an extent, for the effects of noise and volume conduction (40). If there is a consistent phase difference between two signals, commonly referred to as phase synchronization, the value of wPLI tends toward 1; otherwise, it tends toward 0 (Equation 3).

(3) $$\mathrm{wPLI}=\;\frac{\left|E\left(Im\left(e^{i\Delta \theta }\right)\cdot \left|Im\left(e^{i\Delta \theta }\right)\right|\right)\right|}{E\left(\left|Im\left(e^{i\Delta \theta }\right)\right|\right)}$$

Where Δ*θ* is the instantaneous phase difference computed using the Hilbert transform of the signals, and $Im\left(e^{i\Delta \theta }\right)$ is the imaginary part of the complex phase difference.

#### 2.7.9 Band power of gut signals

The peak band power for the filtered gut signal was computed using the pwelch function in MATLAB with a 1-min window and 50% overlap. The peak frequency corresponds to the frequency at which the peak power is observed.

#### 2.7.10 Phase–amplitude coupling between EEG and EGG—a measure of gut–brain coupling

The collected EEG and EGG signals were phase-locked at the level of seconds, and the analytical signal was constructed using the instantaneous amplitude of the higher-frequency EEG signal and the instantaneous phase of the lower-frequency EGG signal. The instantaneous amplitude was computed using the absolute value of the Hilbert transform of the EEG signal, and the instantaneous phase was computed using the angle of the Hilbert transform of the *Z*-score-normalized, filtered EEG signal. Phase amplitude coupling (PAC) for the analytical signal was computed using the following formula (Equation 4),

(4) $${\mathrm{PAC}}_{\mathrm{EEG},\mathrm{EGG}}\;=\;\frac{\sum_{t=1}^{T}|A_{t}^{\mathrm{EEG}}*e^{i\phi_{t}^{\mathrm{EGG}}}|}{\sqrt{T}*\sqrt{\sum_{t=1}^{T}{\left(A_{t}^{\mathrm{EEG}}\right)}^{2}}}$$

The PAC measure was computed using the time points corresponding to the top 5 percentiles of instantaneous amplitude to represent gut–brain coupling.

### 2.8 Feature scrutiny for the prediction model

The extracted features were tested for interfeature correlation. Highly correlated features (> 0.8) were merged by taking their average, and the final set of features was subjected to criteria-based scrutiny, as described in the following section. Data points from participants with missing visits were also included in this process. The values of these feature groups closely approximated the first principal component, with Pearson *corr > 0.98.*

#### 2.8.1 Criterion (i): Does the feature show differences between healthy and depressed individuals?

The extracted electrophysiological features from the baseline visit recordings were tested for statistical differences between healthy and depressed populations based on mental health scores (PHQ-9, healthy: baseline PHQ-9 ≤ 5, depressed: baseline PHQ-9 > 5). If the *p*-value was less than 0.05, the feature met the criterion.

#### 2.8.2 Criterion (iia): Does the change in feature value over time reflect mental health severity?

The change of the electrophysiological feature values at the intermediate follow-up visit from baseline (visit 2–visit 1) was correlated with the change in PHQ-9 scores at the final follow-up visit from baseline (visit 1–visit 3). If the correlation coefficient was significant (*p*-value< 0.05), the feature met the criterion.

#### 2.8.3 Criterion (iib)

In the same line as before, the change in any feature value at the final follow-up visit from baseline (visit 3–visit 1) was correlated with the change in PHQ-9 scores at the final follow-up visit from baseline (visit 1–visit 3). If the correlation coefficient was significant (*p*-value< 0.05), the feature was considered to meet the criterion.

The features that satisfy one of the above criteria were selected for predictive modeling. For baseline features, if criterion (i) is satisfied, the baseline electrophysiological feature was used for model development. On feature plasticity, if criterion (iia) or criterion (iib) is satisfied, the change in feature value at the intermediate follow-up visit from the baseline visit (visit 2–visit 1) was considered.

The final set of features used for predictive model development is presented in **Supplementary Table SA**.

### 2.9 Feature generation for the prediction models

The criteria-based, scrutinized quantitative electrophysiology features, along with demographics of and individual responses to questionnaires such as PHQ-9, GAD-7, and MMSE recorded at visits 1 and 2, were used as input for developing the predictive models. For the electrophysiological data segmented into 1-min fragments, the corresponding demographic variables and subject-level scores were repeated across all fragments. Fragments from the different tasks were concatenated such that the first minute of a given task was concatenated with the first minute of another task.

#### 2.9.1 Feature imputation

For feature categories such as baseline EEG, baseline EGG, change in EEG, and change in EGG, missing data involving fewer than two tasks or shorter task segments were imputed using other features within the same category via the Multiple Imputation by Chained Equations algorithm. Data imputation was applied to a small portion of the dataset (3.4% of total registered subjects, *N* = 7). Specifically, six of these participants required imputation for one task, and one participant required imputation for two tasks. Imputation was performed using site 1 data only, and no imputation was applied to the holdout site data. Missing data included the following: one subject’s visit 2 breathing task data; three subjects’ visit 2 photic task data; one subject’s visit 1 breathing data; one subject’s visit 1 photic data; and one subject’s breathing and photic task data at visit 2. The IterativeImputer function from the scikit-learn library in Python was used, in which values were iteratively imputed in a round-robin fashion for a maximum of 10 iterations until reaching asymptotic stability. Features were generated for five fragments in any task; the average interfragment correlation across features and groups was 0.77 ± 0.19. The IterativeImputer, upon internal testing and cross-validation, yielded an *R*-squared coefficient value > 0.5 and was therefore selected. More details regarding the efficacy of the imputer are provided in the **Supplementary material**.

### 2.10 Stepwise testing of feature categories for predicting treatment response

In order to illustrate the significant incremental variance explained by longitudinal and gut–brain coupling features in addition to baseline EEG, selected longitudinal EEG and gut–brain coupling (PAC) feature groups were used to predict the change in PHQ-9 score from baseline to the final follow-up visit (visit 3–visit 1) using ridge regression. As baseline EEG and scores are readily obtainable in clinical settings, they were tested first. Given the abundance of studies and the accessibility of EEG in clinical settings, longitudinal EEG was added next. Finally, baseline and longitudinal gut features were added to the input features. For each category, principal component analysis was performed to reduce the dimensionality, and the components required to explain 70% of the variance were used as input to ridge regression.

### 2.11 Model outcome operationalization

Five models were developed to compare the performance and explainability of treatment outcome prediction. The model outcomes were classified as patients with SC, NC, or healthy controls. A participant was considered to show a SC if there was ≥ 30% reduction in the final follow-up PHQ-9 score compared with the baseline visit, or if the final follow-up (visit 3) PHQ-9 score was ≤ 5. Such a reduction indicated an improvement in mental health status. The ≥ 30% reduction criterion was used to maintain class balance for training and testing the machine learning models and to account for early assessment of change within just a week. The threshold was therefore chosen for operational and methodological considerations, in contrast to the standard clinical routine of assessing response of > 50% over a 4–6-week timeline from the start of the treatment, which may be too stringent for an early prediction study. If this criterion was not met, the outcome was treated as no change in mental health, and the participant was classified as a “NC”. Healthy controls were defined as nonclinical participants with baseline PHQ-9 scores ≤ 5. Clinically, SC should be interpreted as indicating early meaningful improvement and not as a full clinical response as defined in standard clinical trials.

Models 1 and 2 are multilayer perceptrons that use all scrutinized electrophysiological features, along with demographics and questionnaires, and output whether the subject shows no change in mental health/significant change in mental health (model 1) and NC/SC/HC classifications (model 2), based on baseline PHQ-9 scores and changes in PHQ-9 scores between the baseline visit and the final follow-up visit. The multilayer perceptron (MLP) was built with two hidden nodes using a tanh activation function and a constant learning rate of (alpha = 0.001). The parameters were finalized using a grid search algorithm.

The groups are categorized as HC/SC/NC and are used for model development and analysis. The rows indicate distinct sites of participant recruitment, while the first column indicates the baseline (visit 1) PHQ-9 scores, and the second and third columns indicate the final follow-up (visit 3) PHQ-9 scores.

Model 3 is a logistic regression model that outputs whether a depressed subject is a SC/NC. For the development of this model, only data from subjects whose baseline PHQ-9 score is > 5 were used. Therefore, all healthy controls were excluded, and only two classes were used in this model.

Models 4 and 5 are recurrent neural networks that learn the temporal dynamics distinguishing different signature subject groups, with inputs consisting of 1-min fragments over time. The networks were designed with two hidden nodes leading to the output layer, with either two output nodes representing NC and SC, or three output nodes representing NC, SC, and HC, respectively.

For model development, data from 48 participants were used for three-class models development, and 28 participants comprising only SC and NC were used for two-class model development. The holdout sample consisted of 22 participants with valid input features and available ground-truth labels.

Oversampling and the Synthetic Minority Oversampling Technique (SMOTE) were used across all models to address class imbalance and increase the number of data points in the training dataset, respectively. The imblearn library in Python was used with default parameters, including sampling_strategy as “auto” and n_neighbors set to five. Only the minority classes were oversampled to match the number of samples in the majority class.

### 2.12 Model performance evaluation

The performance of the stepwise feature-category addition model was assessed using a leave-one-subject-out cross-validation strategy. The primary predictive models forming the core of this holistic research approach (Section 2.11) were evaluated using a stratified fivefold cross-validation strategy. Cross-centric holdout validation was performed to assess the generalizability of these models.

### 2.13 Baseline questionnaire-based clustering of phenotypes using unsupervised K-means

The distribution of baseline questionnaires (PHQ-9, GAD-7, MMSE) was presented in a reduced-dimensional form using the K-means clustering algorithm with the number of clusters set to three (optimal clusters identified using elbow and silhouette methods). Each cluster was labeled as HC, SC, or NC if the corresponding data samples formed a two-thirds majority within the cluster.

### 2.14 Supervised subtyping based on symptoms

Furthermore, we clustered the phenotype presentations of participants into gut-symptom or homeostatic regulation-dominant presentations, cognitive-behavioral-dominant phenotypes, and arousal-related presentations. Specifically, any patient was categorized as symptom-dominant if their symptom score was greater than the population median.

Sleep score was computed as the mean of PHQ-9 questions (q) 3 (*Trouble falling or staying asleep, or sleeping too much*) and 4 (*Feeling tired or having little energy*); appetite was defined as PHQ-9 q5 (*poor appetite of overeating*); negative thoughts about self were defined as PHQ-9 q6 (*feeling that you are a failure or have let yourself or your family down*); social symptoms were computed as the mean of questions 7 (*Trouble concentrating on external things*) and 8 (*Moving or speaking so slowly that other people could have noticed*); and anxiety was computed as the mean score of the GAD-7.

### 2.15 Feature importance

SHAPley value is a measure of the marginal contribution of a feature toward the prediction of a class for a given data point. Depending on the feature value, the SHAPley value increases or decreases, and this relationship is captured by the slope of a linear model fitted between the feature values of all data points and their corresponding SHAPley values. The feature importance score is calculated using the following formula (Equation 6),

(6) $$\text{Feature importance score}=\left|\text{Slope}\right|*\left|{\text{SHAP}}_{\text{SC}}-{\text{SHAP}}_{\text{NC}}\right|$$

In the above formula, the score is maximized when the magnitude of the slope is maximized, and the marginal contribution of the feature increases the prediction of one class (SC or NC) while decreasing the prediction of the other.

### 2.16 Statistics

For all correlation analyses, normality was assessed using the Anderson–Darling test. If the data were normally distributed, correlations were computed using Pearson’s correlation method. If a feature did not pass the Anderson–Darling test, Spearman’s rank correlation was used. Since demographics include categorical variables, a Chi-square test was performed to assess statistical significance.

For feature comparisons based on existing theories, either a *t*-test or a rank-sum test was performed, depending on normality, to compute the *p*-value. Since validation of existing theory was not part of hypothesis testing, no correction was applied to the *p*-values. In phenotyping analyses performed using feature categories deemed important by the predictive model, Bonferroni correction was applied to the *p*-values to account for multiple comparisons.

## 3 Results

Our primary study objective is to assess whether behavioral or gut–brain electrophysiology markers can predict the treatment outcomes as early as 7–10 days after intake of medicine in patients with depression. To answer this, we employed a longitudinal study design and recruited treatment-naïve patients and control subjects. Patients initiated medication intake from the index date at visit 1 to the clinic, returned after 7–10 days for visit 2 experimental procedures, and again after 30–40 days for the follow-up visit 3. These patients were prescribed SSRIs, benzodiazepines, or atypical antidepressants. The age-matched control subjects were recruited from the community outside the clinic and were assessed in the research laboratory. **Figure 2** presents the schematic of the experimental timeline, the study setup, and the CONSORT diagram. Initial patient information, including demographics, medication details, and trauma history during the index visit. During visits 1 and 3, electrophysiological data (EEG, EGG) were acquired during various cognitive tasks, along with administration of the PHQ-9, GAD-7, MMSE, and a clinical interview using the HDRS. During visit 2, EEG and EGG data were collected during cognitive tasks, along with well-being scores. The cognitive paradigms included a simple eyes-open resting state, eyes-closed resting, hyperventilation-based interoception, and photic stimulation, which were administered to all participants across all three visits.

All initial data analyses and model development presented in this manuscript were based on data collected from site 1. The control population had a mean age of 34.3 years (± 12.17), with 51 male and 10 female participants. The patient population has a mean of 35.4 years (± 15), with 73 male and 26 female participants. The signal characteristics (**Figure 2D**) show that, broadly across visits, there are no statistically significant differences in spectral EEG presentations between control and depressed populations when averaged across regions; however, theta power in central regions was significantly greater in the depressed population compared with the control population (*p* = 0.014 [left], 0.045 [right]). The control population had higher normogastric EGG power (*p* = 0.048; **Table 2**).

**Table 2:** Summary of demographics and baseline electrophysiology in the population from site 1 used for model development.

Feature	Control	Depressed	*p*-value
Age	34.3 years (± 12.17)	35.4 years (± 15)	0.496
Handedness^*^	50 right-handed, 10 left-handed	94 right-handed, 5 left-handed	0.049
Gender	51 men, 10 women	73 men, 26 women	0.300
History of trauma^*^	57 no	84 no, 10 yes	0.005
History of antidepressants^*^	1 yes, 59 no	38 yes, 61 no	< 0.001
PHQ-9^*^	4.85 (± 4.26)	12.48 (± 5.2)	< 0.001
GAD-7^*^	4.06 (± 3.67)	10.98 (± 4.46)	< 0.001
MMSE^*^	26.75 (± 3.36)	25.24 (± 2.62)	0.001
EGG normogastric power^*^	0.0061 (± 0.0011)	0.0057 (± 0.0012)	0.048
EGG tachygastric power	0.0031 (± 0.0003)	0.0031 (± 0.0002)	0.881

The statistics indicate the p-value of chi-square tests for Handedness, History of Trauma, History of depression medications, PHQ9, GAD7, MMSE, and t-test of the normogastric EGG power are significantly different between non-clinical (control) and clinical (patient)groups.

The data from site 2 were processed separately using the same methods as those applied to the site 1 data. Importantly, the site 2 data served solely as an independent held-out test set to evaluate the model, thereby assessing the robustness and reliability of the model prediction. The patients from site 2 had a mean age of 38.7 years ± 10 years, were all right-handed, and included 26 male and 19 female participants; nine had a history of trauma, and 24 had a history of antidepressant use. Their baseline PHQ-9 score was 14.6 ± 5.7, GAD-7 score was 11.2 ± 4.2, and MMSE score was 20.47 ± 5.7.

**Figure 3:**
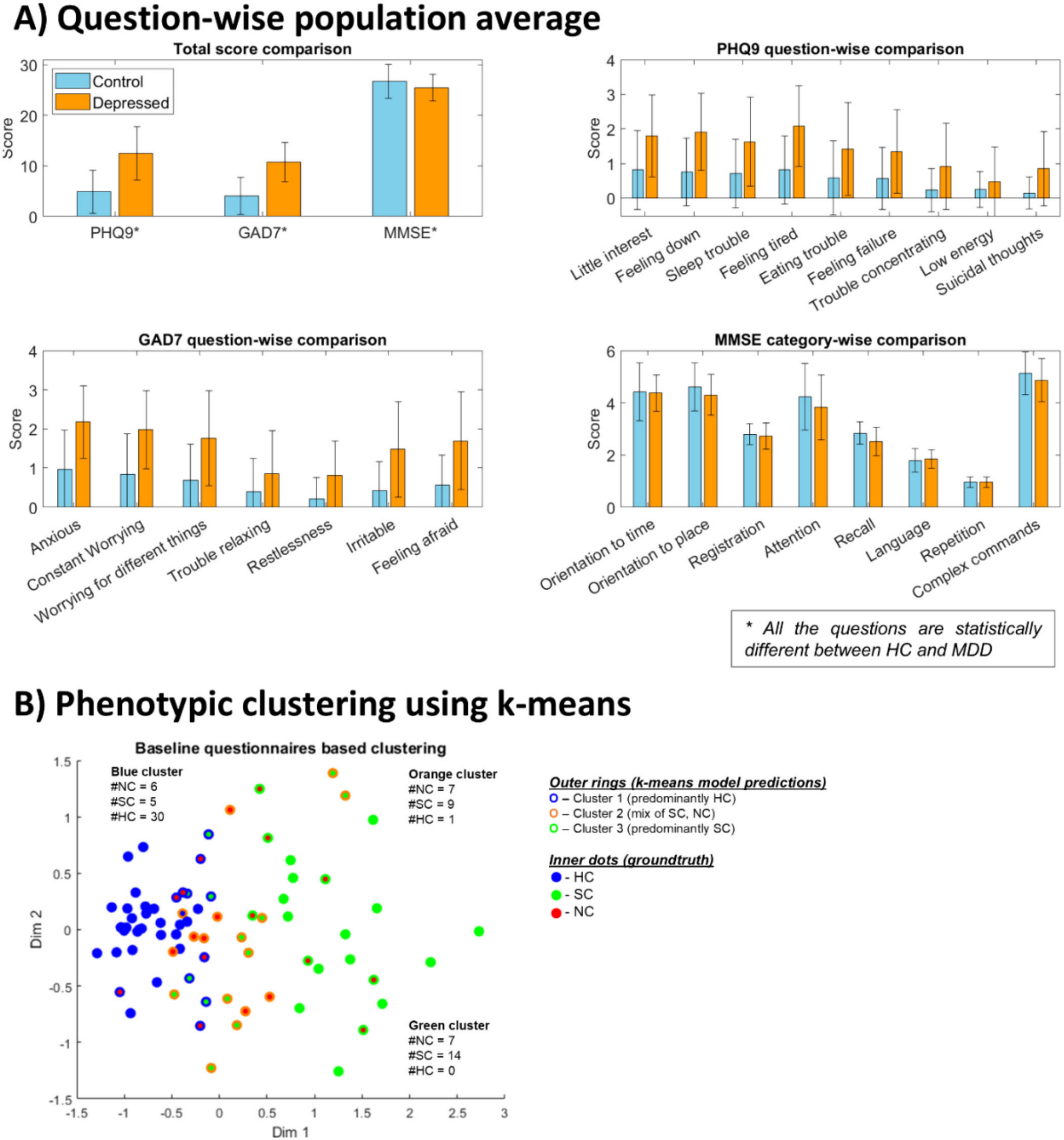
Participants’ clinical characteristics. **(A)** Question-wise comparison of mean scores for the control and depressed populations. **(B)** Baseline questionnaire clustering using k-means algorithm: Phenotypic clustering of information obtained in visit 1 into three groups based on their eventual response characteristics in visit 3 (healthy controls [HC], patients showing significant change in mental health [SC], no change in mental health [NC]), performed using baseline PHQ-9, GAD-7, and MMSE responses. The outline represents the clusters (blue, predominantly HC; orange, mix of SC and NC; green, predominantly SC), and the inner color represents SC (green), NC (red), and HC (blue).

**Figure 3A** presents the initial patient characteristics based on clinical data collected at visit 1. There were significant differences between the control and depressed groups at visit 1 in medication history, trauma history, PHQ-9, GAD-7, and MMSE (**Table 2**). Clinical participants who showed a significant change in mental health (*N* = 28) had slightly higher mean PHQ-9 and GAD-7 scores (question-wise) compared with those who showed no significant change at baseline visit 1 (*N* = 22, refer to **Supplementary Figure 1**). Patients who demonstrated a significant change in mental health response showed improvement across all PHQ-9 and GAD-7 questions.

**Figure 3B** presents the results of baseline phenotypic clustering using questionnaire data, and it was observed that the blue cluster predominantly contained healthy subjects (PHQ-9< 5, 73%), whereas the green cluster represented patients showing significant change in response (67%) and no change in mental health (33%). The orange cluster had an almost equal proportion of responders and nonresponders. These findings suggest that, based on questionnaires, it is difficult to reliably distinguish responders from nonresponders and highlight the need for an objective approach to predict treatment response (*N* = 3 clusters identified as optimal using the elbow and silhouette methods).

### 3.1 Major theories on frontal activation and brain–gut interactions were sensitive to treatment response

In terms of neurophysiological underpinnings, earlier studies suggest at least four main theories for predicting treatment response in depression, such as increased theta cordance and increased magnitude of frontal alpha asymmetry. A decrease in the excitation–inhibition ratio has been reported, suggesting a plausible role of the aperiodic exponent, along with increased gut symptom presentation, indicating a potential role of gut–brain coupling in patients. It was investigated whether these four features—gut–brain coupling, aperiodic exponent, theta cordance, and frontal alpha asymmetry (right–left)—can serve as reliable markers in this study.

At baseline, no significant differences in theta cordance were observed between control and depressed individuals. However, the feature was fairly predictive of early response at visit 2 during eyes-closed cognitive state (*d* = 0.389, *p* = 0.007) and remained reliable at visit 3 (*d* = 0.360, *p* = 0.043), where nonresponders showed greater reduction in frontal theta cordance compared with responders.

**Figure 4:**
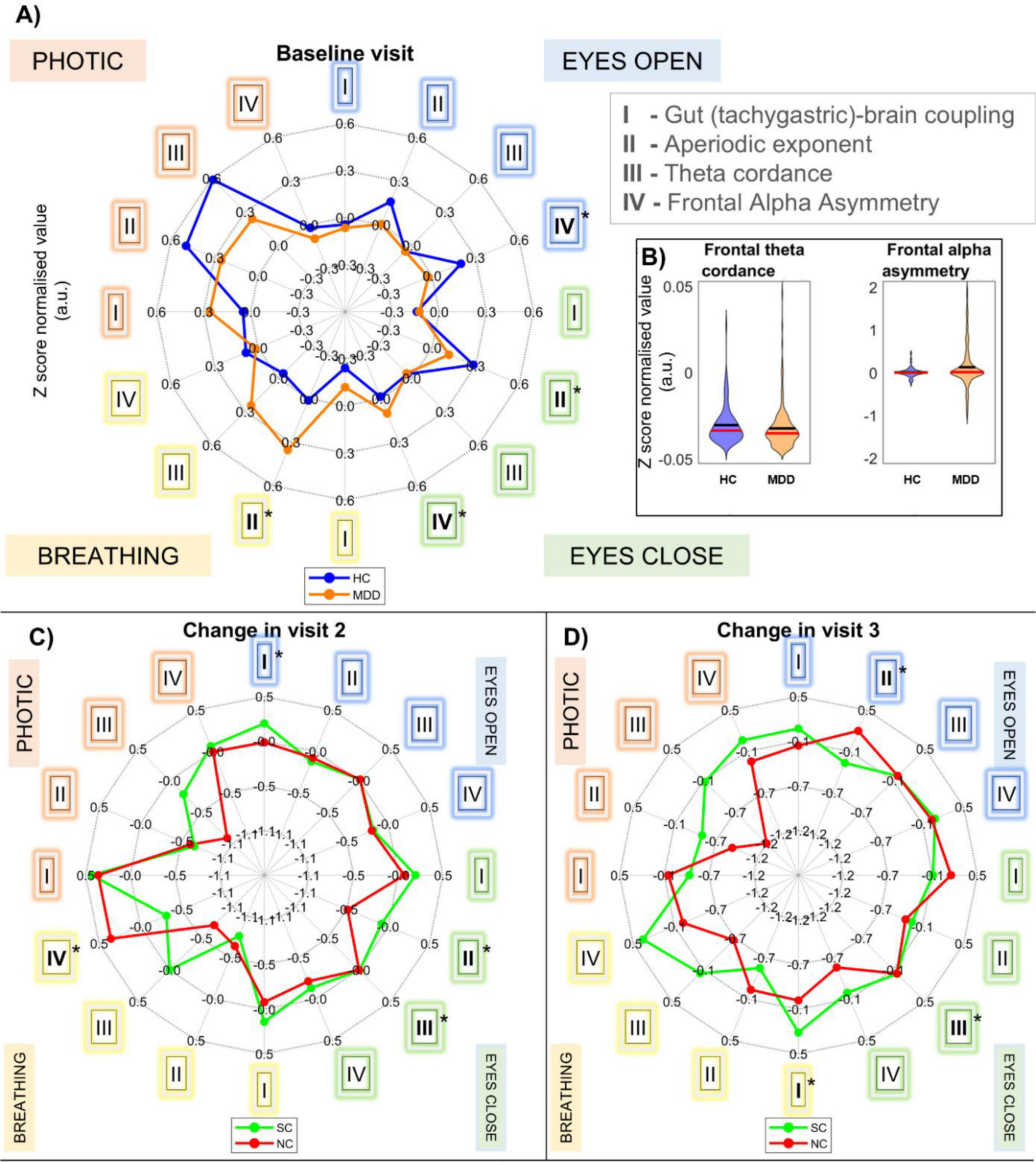
Summary features based on theories of depression. We explore four different theories of depression treatment prediction: increased gut–brain coupling **(I)**, lower aperiodic exponent **(II)**, lower theta cordance (III), and higher frontal alpha asymmetry **(IV)**, when compared to baseline, predict significant response to treatment. Many of these features were sensitive to the cognitive state context to significantly predict the response outcome in patients in baseline visit 1, visit 2, or visit 3. The bolded Greek numerals with asterisk (^*^) represent significant differences in the healthy versus patient samples (baseline) or the responders versus nonresponders (visits 2 and 3) in two-sample hypothesis testing, not controlled for multiple comparisons. At baseline, as per the theories, frontal alpha asymmetry was significantly lower for MDD than HC during the eyes-open condition (*p*-value = 0.0105). The lines in **(A)**, **(C)**, and **(D)** represent the mean of normalized feature values. The average (red line) and median (black line), along with the distribution, are plotted in the violin plot **(B)**.

We also observed greater alpha activation in the right compared with the left hemisphere at baseline in controls than in depressed individuals in the eyes-open resting-state task (*d* = 0.280, *p* = 0.003). Interestingly, in the eyes-closed task (*d* = 0.344, *p* = 0.011), this feature was lower in controls, indicating more balanced right-to-left activations (**Figure 4**), and it was predictive of early treatment response, particularly during interoceptive cognitive state (*d* = 0.593, *p* = 0.004).

The grand-average aperiodic exponent across frontal brain regions was significantly higher in depressed individuals compared with healthy participants during the breathing task (*d* = 0.381, *p* = 0.042). Over time, the feature decreased more in nonresponders, particularly during the eyes-closed state (*d* = 0.328, *p* = 0.048) in visit 2, but showed the opposite trend during the eyes-opened state (*d* = 0.391, *p* = 0.027) in visit 3.

Although there were no significant differences in gut–brain coupling across tasks at baseline, medication increased the coupling value in responders for the eyes-open task at visit 2 (*d* = 0.287, *p* = 0.029) and the breathing task at visit 3 (*d* = 0.749, *p* = 0.033) (**Figure 4**).

The above results present evidence against the reliability of any single feature identified in earlier literature and its predictive ability to classify responders from nonresponders. Subsequently, it was investigated whether multivariate response patterns, rather than a single biomarker, could better characterize treatment response and predict outcomes as early as 1 week. To address these questions, machine learning model analyses were performed in the following section.

### 3.2 Longitudinal designs and gut–brain approach can significantly improve the prediction of responses

How do different forms of data, such as clinical reports, EEG, and EGG collected at different time points during treatment, contribute to response prediction? We begin investigating this question using a ridge regression model in this section and later in the manuscript using various machine learning models. We first specify a simple regression model using only baseline EEG features collected during visit 1. The cross-validated *R*-squared score of the model was 0.4, indicating that it explained about 40% of the variance in response-related changes in PHQ-9 severity at visit 3 in the patient group. Interestingly, adding longitudinal brain information further improved the explained variance to approximately 60%. Incorporating longitudinal changes in both brain and gut signals up to visit 2 explained > 70% of the variance (**Table 3**). Notably, when the model was used to predict PHQ-9 severity at visit 3, baseline features alone explained about 27% of the variance; adding longitudinal EEG increased this to about 38%, and further adding EGG increased it to 51% of the variance in absolute severity scores. These findings guided the development of an explainable prediction model to classify responders from nonresponders using longitudinal electrophysiological data.

**Table 3:** Model selection for predicting treatment outcomes: importance of longitudinal design and brain–gut coupling in characterizing treatment strategies for patients.

Response model	Baseline EEG (r2)	+Longitudinal EEG (r2)	+Longitudinal EGG (r2)
Ridge regression	0.4	0.66	0.71

Ridge regression model performance after including various feature groups in steps.

### 3.3 A perceptron model can efficiently predict early treatment response in patients showing a significant response

Next, we asked whether treatment response can be predicted early at visit 2. Forty-eight participants had complete EEG and EGG data across all tasks for all three visits and were included in the development of the predictive model (**Figure 2C**). We used MLP and simple recurrent neural network (RNN) models to classify patients who would exhibit a significant reduction in mental health severity (SC), no change in mental health (NC), or be healthy controls (HC). The feature selection process yielded electrophysiological features: 12 (baseline) + 11 (longitudinal) from the eyes-open resting-state task, 10 + 22 from the eyes-closed resting-state task, 6 + 14 from the breathing task, and 7 + 12 from the photic administration task (refer to **Supplementary Table 1** for the feature list), along with demographics (5), and questionnaires (24). First, we deployed three-class models, which were validated using *k*-fold cross-validation (*k* = 5), run over 10 instances. The overall cross-validation mean accuracy for MLP was > 74% (~ 37/48), and for RNN was > 70% (34/48), which was well above the chance level of 33% (**Table 4**).

**Table 4:** Model selection for predicting treatment outcomes: based on model comparisons, the two-class MLP is best suited for predicting participants (boldened) with no change in mental health, and logistic regression is more sensitive for predicting significant change in mental health.

Model	No. classes^a^	Accuracy%	Sensitivity% (NC)	Specificity% (SC)
Multilayer perceptron	3	78.3	73.1	76.7
	2	**81**	**84.1**	77.9
Recurrent neural networks	3	72.5	61.4	72.5
	2	75	75.4	74.7
Logistic regression	2	80	80.9	**79.2**

^a^ Classes—3: HC, NC, and SC; 2: SC and NC.

We also tested two-class models specifically trained on patients’ data to early predict whether a patient will show a significant change/no change in mental health by the end of the treatment course. For this question, we compared the performance of the previously described MLP and RNN architectures using a two-class output, along with logistic regression. All three models were trained and cross-validated using 28 subjects from site 1 and performed well above the chance level of 50%. The MLP model outperformed the other two with an accuracy of ~ 81% (23/28), while the accuracy of logistic regression was ~ 79% (22/28), and that of the RNN was ~ 75% (21/28). The sensitivity of the two-class MLP model in predicting a participant with no change in mental health was ~ 84%. The relative risk of our model, computed as the proportion of subjects falsely predicted as nonresponders, was two subjects (16%). Matthew’s coefficient, accounting for all the depressed subjects, was 0.62. The specificity of the model for predicting significant change in mental health was 78% (**Table 5**). Among the five individual models and their nested architecture, the MLP architecture had the lowest relative risk, and the model was selected for further interpretation of the importance of each feature in the prediction using SHAPley values.

**Table 5:** Confusion matrix of two-class MLP model prediction for site 1 validation.

Multilayer perceptron	Predicted		
Actual		NC	SC
	NC	10.6 (± 1.4)	3.4 (± 1.4)
	SC	2 (± 1)	12 (± 1)

Importantly, we also tested the model using independent site 2 data, and the MLP model showed an accuracy of 77.27%, with a sensitivity for nonresponders of 71.43% and a specificity for responders of 80%, confirming the robustness and reliability of our model predictions (**Table 6**).

**Table 6:** Confusion matrix of two-class MLP model prediction for site 2 independent testing.

Multilayer perceptron	Predicted		
Actual		NC	SC
	NC	5	2
	SC	3	12

### 3.4 Feature marginal contribution is sensitive to symptom presentation in patients

**Figure 5:**
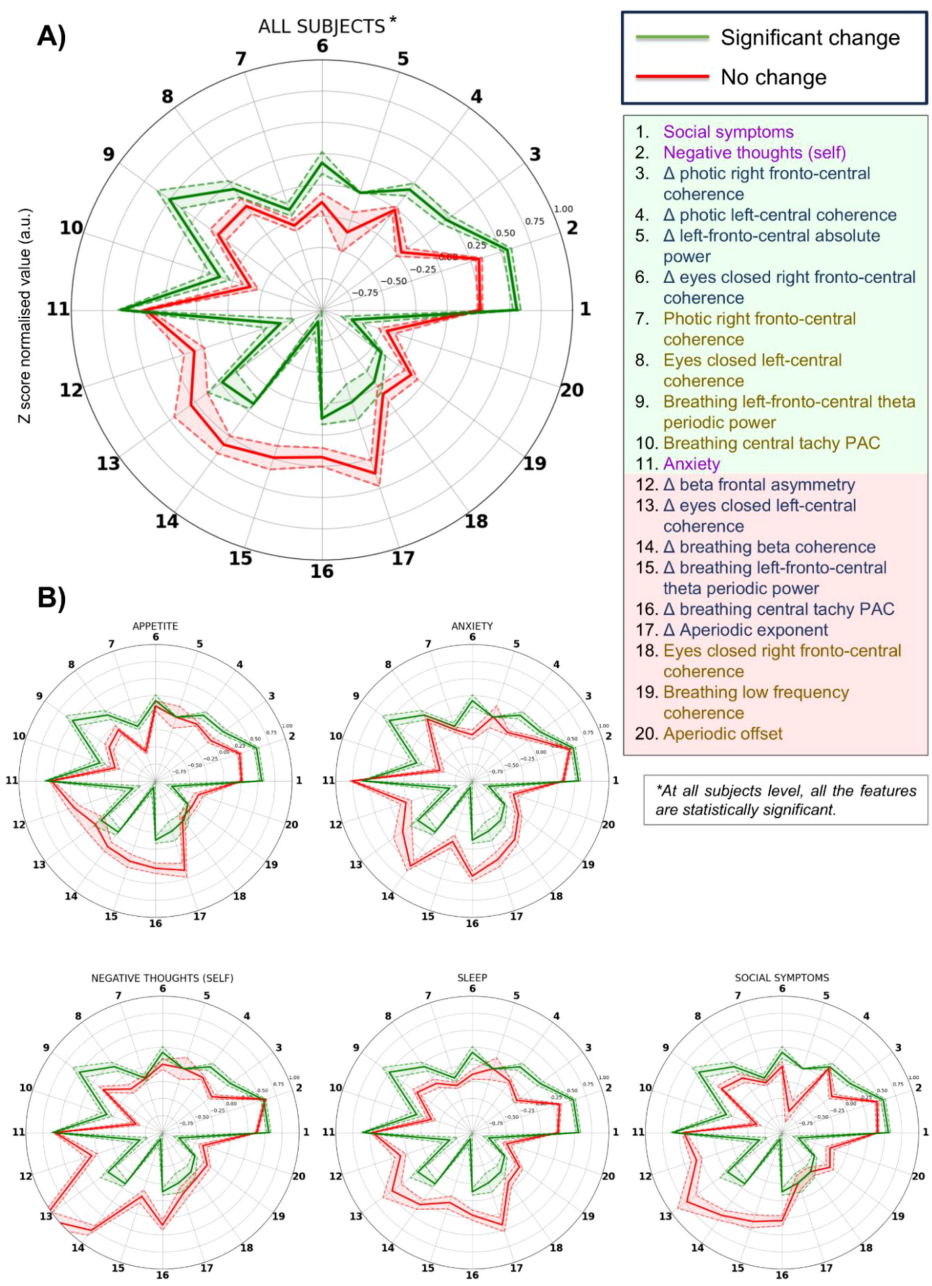
Interpretable and predictive modeling of treatment outcome. **(A)** Z-scored normalized comparison between responders (SC, green) and nonresponders (NC, red) of top features significantly contributing to the multilayer perceptron two-class model performance. The shaded region represents the SEM for *N* = 28; the green legend shading reflects a feature increase for the response, while the red indicates the contrary. **(B)** The schematic feature profiles for selected participants with high severity in appetite, anxiety, negative thoughts about self, sleep, and social symptoms.

We computed feature importance as the marginal contribution of top feature groups that, in sum, explained > 95% of the observed response prediction (**Figure 5**) in the two-class multilayer perceptron. The results suggest that, overall, changes in left frontocentral absolute theta power, global coherence across bands during the photic task, and coherence between regions other than the central left during the eyes-closed task are higher in participants showing a significant change in mental health. On the other hand, changes in coherence during the breathing task, central left coherence during the eyes-closed task, beta asymmetry between hemispheres (frontal), periodic theta power in the left frontal region, aperiodic exponent, and tachygastric gut rhythm–brain coupling decrease.

We also observed differences in features based on the specificity of symptom severity. Longitudinally, in the interoceptive breathing task, global beta coherence and tachygastric gut rhythm coupling with central brain regions were reduced for responders, independent of the symptom manifestation. The aperiodic exponent decreased selectively for responders with high sleep symptom manifestation. Similarly, left frontocentral theta absolute power increased selectively in responders with social symptoms. Longitudinally, a decrease in beta frontal asymmetry and left frontocentral theta periodic power during the breathing task was significantly sensitive to treatment response across all symptom groups except anxiety. Overall, we observed that different electrophysiological biomarkers were predictive of treatment outcomes depending on their subtype (**Table 7**).

**Table 7:** Significant features for specific phenotypic subtypes: electrophysiological feature comparison for responders and nonresponders in a subset of participants with specific symptom severity and symptom scores above the population median.

Feature	Anxiety	Appetite	Negative thoughts (self)	Sleep	Social symptoms
Δ photic right frontocentral coherence (ΔT_4_ Coh F_R_C_R_)	0.064 ± 0.45, 0.181 ± 0.43	0.004 ± 0.45, 0.284 ± 0.5	− 0.109 ± 0.53, 0.131 ± 0.42	− 0.196 ± 0.46, 0.222 ± 0.45	− 0.101 ± 0.51, 0.174 ± 0.4
Δ photic left central coherence (ΔT_4_ Coh C_L_)	− 0.123 ± 0.37, 0.254 ± 0.52	0.029 ± 0.26, 0.331 ± 0.5	− 0.001 ± 0.31, 0.217 ± 0.55	− 0.07 ± 0.3, 0.248 ± 0.52	0.161 ± 0.17, 0.147 ± 0.51
Δ left frontocentral absolute power (ΔF_L_C_L_ Abs power)	− 0.012 ± 0.01, − 0.018 ± 0.02	− 0.018 ± 0.01, − 0.016 ± 0.02	− 0.012 ± 0.01, − 0.016 ± 0.02	− 0.015 ± 0.01, − 0.016 ± 0.02	− 0.675 ± 1.93, − 0.015 ± 0.02
Δ eyes-closed right frontocentral coherence (ΔT_2_ Coh F_R_C_R_)	− 0.323 ± 0.33, 0.377 ± 0.78	0.098 ± 0.47, 0.237 ± 0.84	0.001 ± 0.56, 0.293 ± 0.78	− 0.147 ± 0.66, 0.273 ± 0.75	− 0.03 ± 0.49, 0.216 ± 0.75
Photic right frontocentral coherence (T_4_ Coh F_R_C_R_)	− 0.213 ± 0.24, − 0.214 ± 0.42	− 0.542 ± 0.29, − 0.104 ± 0.37	− 0.171 ± 0.38, − 0.216 ± 0.42	− 0.274 ± 0.39, − 0.215 ± 0.41	− 0.231 ± 0.36, − 0.137 ± 0.42
Eyes-closed left central coherence (T_2_ Coh C_L_)	0.119 ± 0.61, 0.259 ± 0.67	− 0.074 ± 0.67, 0.345 ± 0.77	− 0.211 ± 0.39, 0.263 ± 0.65	− 0.108 ± 0.56, 0.241 ± 0.71	− 0.012 ± 0.64, 0.264 ± 0.65
Breathing left frontocentral theta periodic power (T_3_ F_L_C_L_ θ Per power)	− 0.34 ± 0.41, 0.207 ± 0.89	− 0.16 ± 0.43, 0.7 ± 1.15	0.072 ± 0.98, 0.367 ± 1.1	0 ± 0.71, 0.398 ± 1.02	0.095 ± 0.86, 0.521 ± 1.05
Breathing central tachypnea (T_3_ Cen-Tachy PAC)	− 0.501 ± 0.46, − 0.237 ± 0.72	− 0.376 ± 0.62, − 0.106 ± 0.82	− 0.593 ± 0.45, − 0.186 ± 0.7	− 0.368 ± 0.59, − 0.189 ± 0.74	− 0.609 ± 0.45, − 0.188 ± 0.66
Δ beta frontal asymmetry (Δβ Fr Asym)	0.021 ± 1.17, − 0.331 ± 0.81	0.259 ± 0.71, − 0.612 ± 1.12	0.09 ± 1.06, − 0.398 ± 0.83	0.105 ± 0.83, − 0.588 ± 1.04	0.315 ± 0.51, − 0.48 ± 0.79
Δ eyes-closed left central coherence (ΔT_2_ Coh C_L_)	0.261 ± 0.29, 0.063 ± 1.24	0.087 ± 0.39, 0.249 ± 1.33	1.154 ± 1.95, 0.032 ± 1.26	0.453 ± 1.59, 0.11 ± 1.2	0.723 ± 1.76, − 0.106 ± 1.15
Δ breathing beta coherence (ΔT_3_ β Coh)	0.543 ± 0.78, − 0.026 ± 0.47	0.192 ± 0.12, − 0.278 ± 0.33	0.772 ± 0.79, − 0.054 ± 0.47	0.305 ± 0.79, − 0.08 ± 0.45	0.501 ± 0.56, − 0.039 ± 0.41
Δ breathing left frontocentral theta periodic power (ΔT_3_ F_L_C_L_ θ per power)	− 0.066 ± 0.93, − 0.586 ± 0.43	0.235 ± 0.71, − 0.969 ± 0.82	− 0.018 ± 0.84, − 0.623 ± 0.47	0.078 ± 0.75, − 0.825 ± 0.75	0.37 ± 0.91, − 0.753 ± 0.5
Δ breathing central tachy PAC (ΔT_3_ Cen-Tachy PAC)	0.394 ± 0.74, − 0.113 ± 0.4	0.282 ± 0.6, − 0.176 ± 0.38	0.356 ± 0.61, − 0.143 ± 0.39	0.21 ± 0.67, − 0.111 ± 0.42	0.291 ± 0.56, − 0.13 ± 0.39
Δ frontocentral aperiodic exponent (ΔAp exponent)	0.209 ± 1.09, − 0.294 ± 1.34	0.377 ± 1.03, − 0.299 ± 1.41	− 0.049 ± 1.09, − 0.214 ± 1.36	0.422 ± 1.02, − 0.322 ± 1.3	− 0.228 ± 0.68, − 0.167 ± 1.24
Eyes-closed non-left-central coherence (T_2_ Coh F_R_C_R_)	0.058 ± 0.38, − 0.342 ± 0.76	− 0.33 ± 0.57, − 0.054 ± 0.92	− 0.167 ± 0.53, − 0.318 ± 0.76	− 0.1 ± 0.66, − 0.374 ± 0.72	− 0.29 ± 0.44, − 0.251 ± 0.86
Breathing low-frequency coherence (T_3_ Coh θ, α)	− 0.164 ± 0.5, − 0.347 ± 0.37	− 0.348 ± 0.54, − 0.416 ± 0.37	− 0.193 ± 0.46, − 0.362 ± 0.37	− 0.147 ± 0.54, − 0.393 ± 0.37	− 0.135 ± 0.4, − 0.383 ± 0.34
Frontocentral aperiodic offset (Ap offset)	− 0.366 ± 0.68, − 0.917 ± 0.72	− 0.339 ± 0.56, − 0.648 ± 0.79	− 0.381 ± 0.64, − 0.94 ± 0.71	− 0.471 ± 0.65, − 0.901 ± 0.67	− 0.263 ± 0.45, − 0.73 ± 0.75

The above table highlights feature groups from the top 20 that differ significantly between responders and nonresponders. Cells highlighted in green indicate a higher mean in SC compared with NC, whereas red cells indicate the opposite; both suggest that the feature response may be associated with the criteria defining a specific phenotypic subtype (column index). Cells display the mean and standard deviation of NC and SC data points. *A Bonferroni-corrected *p*-value< 0.05 was marked as significant. Phenotypes were constructed as follows: sleep score = mean of PHQ-9 q3 and q4; appetite = PHQ-9 q5; negative self-thoughts = PHQ-9 q6; social symptoms = mean of PHQ-9 q7 and q8; anxiety = mean GAD-7 score.

Notably, severe anxiety symptoms have been shown to have a lower remission rate in larger multicentric international studies (41). Drawing on the Research Domain Criteria (R-DoC) framework for dissociable neural circuit mechanisms, as well as the Tripartite Theory of Depression (Anna 42), we clustered phenotype presentations in our participants into gut symptoms or homeostatic regulation-dominant symptom presentations, cognitive-behavioral-dominant phenotypes, and arousal-related presentations. These are suggested to have, to some extent, distinct circuit underpinnings, such as vagal/gut signaling, cognitive and action execution circuit dynamics, and general arousal indicators, respectively.

**Figure 6:**
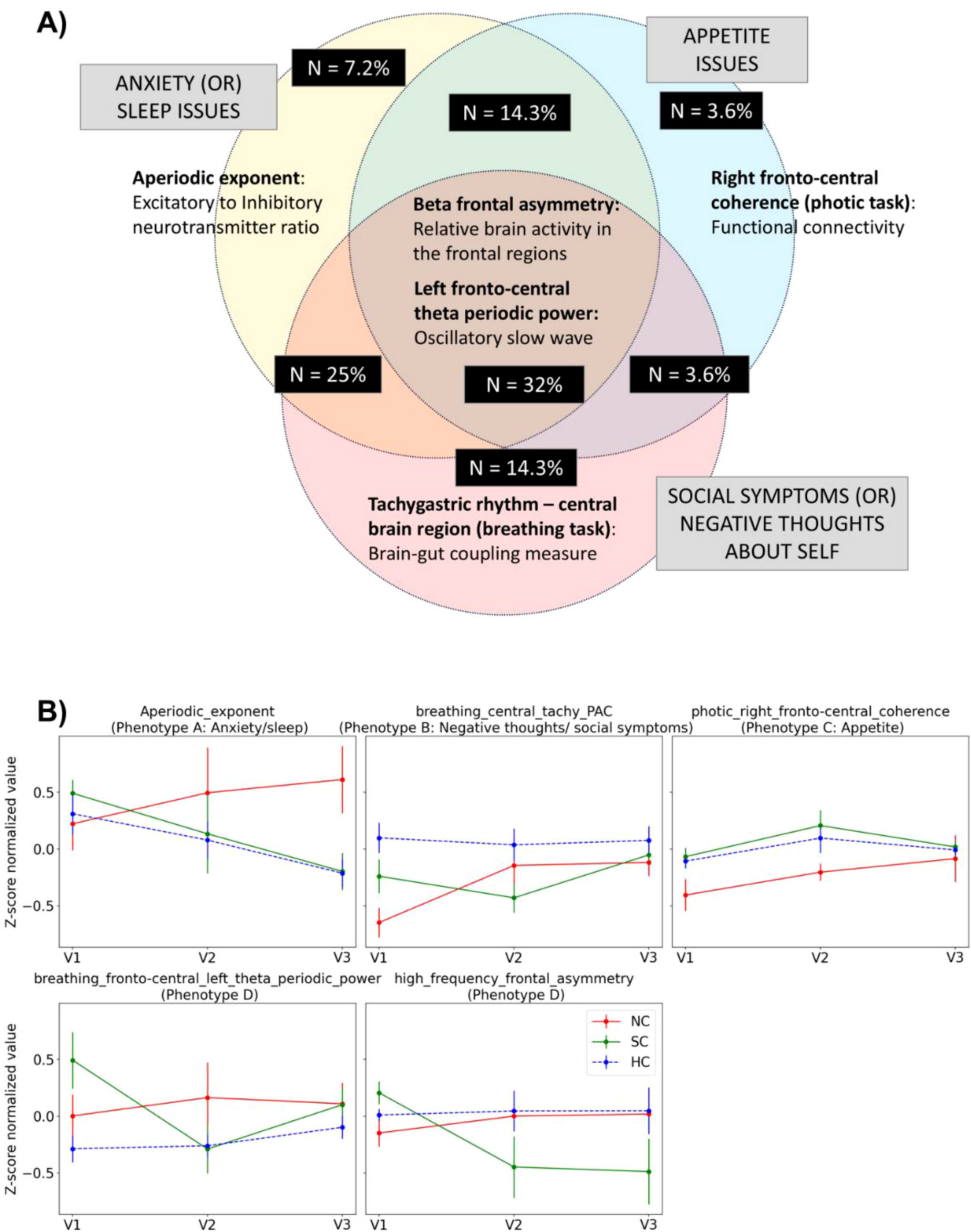
Phenotypic subtype changes through time. **(A)** Phenotypic subtypes in our patient population are represented as a Venn diagram. The diagram shows the proportion of patient population having higher than median severity of the phenotypes, indicating dominant sleep or anxiety symptom presentation, gut or homeostatic regulation symptoms, negative thoughts about self-symptoms, and their combinations. **(B)** Longitudinal group-level changes for top Shapley value features from each phenotype. The representative features were chosen from **Table 7**, and the same have been plotted across time points (V1, V2, V3) for no change, significant change, and healthy controls. The error bars represent SEM. Note: The plot aims to represent the desired direction of change, and all the features are significantly different for SC vs. NC, as mentioned in previous sections.

We then group participants’ symptom profiles into three main categories: gut or homeostatic issues, cognitive-behavioral symptoms, and arousal-related symptoms (**Figure 6**). A participant is considered dominant in a category if their score exceeds the population median. Sleep is measured as the average of PHQ-9 questions 3 and 4, appetite from question 5, negative self-perception from question 6, social symptoms from the average of questions 7 and 8, and anxiety from the average GAD-7 score. We observed *N* = 7 participants belonging to pure phenotypic subtypes (arousal and anxiety, appetite and homeostatic regulation, self-referential processing, and suicidal cognition), *N* = 12 presenting a combination of phenotypic subtypes, and *N* = 9 presenting all three phenotypic subtypes. The presence of a symptom was dichotomized based on the median value of baseline PHQ-9 questions. Participants with scores greater than the median value were considered to manifest the symptom.

For participants with anxiety or sleep issues, the aperiodic exponent significantly decreases across visits in responders (SC), while it remains the same in nonresponders (NC) (*p*-value< 0.001). When a similar analysis was performed for participants with negative thoughts about themselves, we observed that the representative feature, i.e., coupling between tachygastric gut rhythm and central brain regions, showed a sudden increase in nonresponders postmedication (*p*-value = 0.006) and remained consistently elevated across time points. For participants exhibiting appetite or homeostatic dysregulation symptoms, coherence in right frontocentral regions during the photic administration task remained consistently high from baseline (*p*-value = 0.042) to visit 2 (*p*-value = 0.031) in responders compared with nonresponders. Notably, the significant responder profile closely followed the expected change in the control population. Participants exhibiting composite symptom presentation showed a decrease in left frontocentral theta periodic power (*p*-value = 0.003) and beta frontal asymmetry (*p*-value = 0.015) in responders. The reduction in beta frontal asymmetry was consistently seen across time points in responders; however, it was not observed in the healthy controls (*p*-value = 0.013).

## 4 Discussion

### 4.1 Current “watchful waiting” treatment approaches

Treatments for depression, such as medication, supportive therapies, and interventions such as magnetic stimulation or cognitive restructuring, rely heavily on continuous monitoring to predict treatment outcomes. However, with the increasing patient-to-clinician ratio, accurately predicting treatment efficacy through traditional in-person interviews has become increasingly difficult. More than one-third of patients discontinue treatment within the first 30 days due to intolerable side effects. If depression persists after two different medication trials, the patient is often considered treatment-resistant. The “watchful waiting” approach, in which clinicians observe over several weeks to determine whether a treatment is effective, has been criticized for its inefficiency and high resource consumption. Studies show that fewer than 20% of patients benefit from this method (1–4, 23, 43). Given these challenges, there is an urgent need for tools that can predict treatment outcomes earlier—within 2 weeks—enabling clinicians to adjust strategies before patients experience prolonged suffering or side effects (44, 45).

Our study addresses this gap by showing that early predictions of treatment outcomes in depression can be made within just 2 weeks by analyzing electrophysiological markers in both the brain and gut, with 77.27% test accuracy. Specifically, our study identifies early markers that were significantly predictive of treatment response as early as 7–10 days from the start of treatment. Gut–brain connectivity, a novel aspect of our approach, provided additional early markers, pointing to the utility of incorporating gut dysfunctions alongside brain measures. These findings underscore the importance of integrating gut–brain physiology into treatment predictions, which allows clinicians to intervene much earlier in the treatment process.

An important aspect of our study is the use of phenotypic subtyping—identifying depression subtypes based on symptom profiles (e.g., anxiety, sleep disturbances, appetite issues, negative self-thoughts, or social withdrawal)—to predict treatment outcomes. Our results suggest that personalized predictive markers should be tailored to each subtype for more accurate and efficient outcomes. For instance, we found that increased frontocentral excitation–inhibition ratios were the strongest predictors of treatment response in patients with dominant anxiety or sleep-related symptoms. In contrast, decreased tachygastric coupling predicted a positive response for patients with negative self-thoughts. Additionally, right frontocentral connectivity was associated with positive treatment outcomes in patients experiencing appetite-related issues.

### 4.2 Both periodic and aperiodic activations predict treatment outcome

Frontal changes in various frequency bands have been identified as markers of depressive symptoms and treatment response in numerous studies. Some specific markers include increased theta cordance in depressed individuals, especially in the frontal region (46). However, contradictory viewpoints have been reported regarding the oscillatory mechanistic underpinnings, as separation of theta power into its aperiodic and periodic components suggests that the aperiodic component more strongly reflects depression measures relatively (22). These conflicting findings raise questions about the underlying processes mediating depression severity and its response to treatment. We disentangled the periodic components of oscillations to specifically examine the role of periodic theta components in predicting response to medication. Interestingly, periodic theta power in the left frontal and central regions reduced postmedication, as reported in previous literature. On the other hand, aperiodic parameters such as slope and offset played a crucial role in categorizing individuals as responders/nonresponders, highlighting the importance of aperiodic parameters as biomarkers for treatment outcome prediction. Aperiodic offset and aperiodic exponent are measures of global signal power and excitation–inhibition (E/I) neurotransmitter ratio. A greater offset and a lower exponent value indicate balanced power between frequency bands and increased global power. A steeper exponent indicates greater low-frequency power compared with high-frequency power and is commonly observed in a state of relaxation. Antidepressants such as SSRIs and SNRIs are reported to increase the global band power of the signal.

### 4.3 Frontal beta asymmetry can early predict treatment outcome

The slowness of brain activity—in other words, the power in the lower-frequency spectrum—has been related to treatment response in many studies, including within the alpha band. Frontal alpha asymmetry between the left and right hemispheres has, in particular, been identified as a signature of depression severity. Studies suggest that asymmetry in the brain facilitates differential responses to positive versus negative affect, with reduced responsiveness to positive affect and increased responsiveness to negative affect (47, 48). However, frontal alpha asymmetry has not been consistently observed as an indicator of treatment response but rather as a marker of depressive experience (21). The affective responses elicited by positive or negative stimuli have also been separately studied by several researchers to investigate emotional sensitivity in relation to depression severity and treatment response; however, no robust results have been reported (19, 49). In our study, we did not observe a strong effect of frontal alpha power in predicting response. Interestingly, frontal beta asymmetry was found to be sensitive to treatment response, and beta power in the left frontal region relative to the right frontal region increased significantly in depressed individuals who responded to medication. Some studies have specifically examined reward processing and inhibitory control mechanisms to better understand depression severity (50, 51) and response to treatment (52). Not surprisingly, evidence points to a significant presence of reward information in the frontal beta oscillations, suggesting their potential role in early prediction of outcomes (53).

### 4.4 Brain connections and their gut modulatory indices hold important information on treatment effects

Recently, the role of connectivity has garnered increased attention (Elam et al., 2021), and reduced connectivity has been reported in individuals experiencing depression (54, 55). Structural-level investigations have also suggested the possibility of short-range excitation and long-range reductions in connections in depression (56). In our study, we found a significant contribution of connectivity features across theta, alpha, and beta bands in predicting treatment response.

Our study is the first to test the role of gut–brain coupling in explaining depression severity and response to treatment. Interestingly, we found it to predict treatment response as early as 2 weeks after treatment onset. The role of gut dysfunction in depression has been investigated by numerous studies. To our knowledge, gut dysfunction manifested as abnormal motility of the stomach and intestine has not yet been explored in treatment stratification for depression. Our study strongly suggests that examining gut–brain coupling may help optimize personalized treatment strategies. Importantly, it highlights that a scalable electrogastrography tool could be used to compute gut–brain coupling. In our population, we observed that brain–gut coupling in the tachygastric frequency range was significantly reduced in responders following medication.

Furthermore, our study also proposes that a longitudinal design capable of tracking plasticity in neural circuits explains depression measures more effectively than a cross-sectional design that relies on studying and developing prediction models based on baseline time samples in isolation. This is consistent with many studies reporting greater sensitivity of longitudinal markers in depression (16, 57). More importantly, the connectivity and coupling features discussed in this study broadly reflect correlations between two regions or signals (e.g., PAC and coherence) and primarily capture statistical associations or synchrony between signals rather than direct physiological or causal mechanisms. Accordingly, these features should be interpreted as correlational markers that may have clinical relevance but do not, on their own, establish causal brain–gut pathways underlying depression or treatment response.

### 4.5 Cognitive control and sensitivity of task paradigms in treatment response

An important finding in our study is the sensitivity of cognitive control during the administration of specific task paradigms, which serve as reliable markers of treatment response. Beyond traditional resting-state paradigms (eyes open and eyes closed), we identified highly predictive markers during photic flicker presentation and hyperventilating breathing tasks. The photic flicker effect, which is known to modulate cognitive processes depending on stimulus frequency, can influence how external stimuli or internal thoughts are processed, especially through phase coupling with flicker onset (58, 59). Similarly, deep breathing paradigms, which have previously been shown to be sensitive to depressive symptoms, were also found to be valuable for assessing treatment response in our study (60, 61).

### 4.6 Phenotype-based analysis of treatment response mechanisms

Analyzing phenotype-based features for treatment response offers valuable insights into the distinct neurophysiological mechanisms underlying each depression subtype. Our findings identify specific markers for each subtype, which can inform more targeted treatment strategies.

#### 4.6.1 Phenotype A: anxiety/sleep

Dysfunction of the locus coeruleus (LC) has been implicated in depression, leading to reduced norepinephrine levels in the system, which play a key role in the sleep/wake cycle and arousal (62, 63). Electrophysiologically, LC dysfunction manifests as reduced coherence, increased aperiodic exponent, and altered excitatory/inhibitory (E/I) neurotransmitter ratios. In participants with dominant anxiety and sleep issues, we observed reduced coherence during photic and eyes-closed tasks, which increased after treatment. Additionally, the aperiodic exponent in the frontocentral region decreased, indicating a shift toward a more balanced E/I ratio. We propose that interventions (ex. 64) regulating the overall frontocentral E/I ratio may specifically help in addressing this subtype-related symptoms.

#### 4.6.2 Phenotype B: negative thoughts about self/social symptoms

Depression significantly impairs self-referential cognition, which is closely linked to the default mode network (DMN), a network active during resting states and involved in processing self-related information (65). Hyperactivity of the DMN is often associated with rumination, a hallmark of depression in which individuals engage in negative self-reflection (66). In our study, participants with negative self-thoughts or social symptoms showed increased left frontocentral power after treatment, which we interpret as a potential marker of DMN regulation. Moreover, brain–gut coupling, particularly in the somatosensory-motor region during interoceptive breathing tasks, was significantly disrupted in these individuals but improved posttreatment. Interestingly, earlier studies support somatosensory-motor sensitivity and its activation as a significant marker of intervention for suicidal thoughts (67). We propose that interventions should specifically focus on decreasing the activity of the DMN (e.g., rTMS, 66) and improving the sensorimotor activation in response to interoceptive signals to address this subtype characterized by increased negative thoughts about oneself.

#### 4.6.3 Phenotype C: appetite

The insula plays a central role in regulating gastric motility and receives afferent inputs from the nucleus tractus solitarius (NTS), which is involved in processing visceral sensory information from the vagus nerve (68). Dysfunction in these regions can result in irregular gastric activity, often manifesting as appetite issues in depression. In our study, we found decreased right frontocentral connectivity in participants with appetite-related symptoms during photic stimulation, which may be linked to underlying insular dysfunction. The NTS, which is responsible for glutamate release and relaying vagus nerve signals, may be central to this process. We propose that vagus nerve stimulation could be an effective treatment for individuals with this phenotype, improving homeostatic regulation and appetite-related symptoms.

#### 4.6.4 Phenotype D: composite symptom profile

For patients with a composite symptom profile, in which symptoms span appetite, anxiety, sleep disturbances, and negative thoughts, we observed highly sensitive markers during interoceptive breathing tasks. Theta periodic power in the left frontocentral region was elevated at baseline but decreased after treatment in responders. Additionally, frontal beta asymmetry, characterized by increased activity in the left frontal region, served as a key predictor of treatment response. Given the complex nature of this phenotype, a multimodal treatment strategy—drawing from approaches used for the more distinct subtypes—may be most effective for addressing the combined symptoms in this group.

### 4.7 Check for information leakage in our methods

The features that significantly explained the response across the entire dataset were highly correlated with those observed in a subset comprising 80% of the population (Pearson’s *corr* = 0.9), ruling out information leakage in our results. Additionally, we ensured that the training and test datasets do not overlap and employed a *k*-fold cross-validation approach to further validate our findings.

### 4.8 Summary

Overall, our study demonstrates that integrating brain and gut data—along with phenotypic subtyping—can reliably predict a patient’s likelihood of responding to treatment within just 2 weeks. This represents a significant improvement over the standard 4–6-week assessment period, offering clinicians the ability to adapt treatment strategies more quickly and effectively. By understanding the relationship between neural and gut circuits in different phenotypic subtypes, we can provide a personalized precision medicine approach that aligns treatment strategies with specific symptom profiles, ultimately improving patient outcomes and minimizing unnecessary side effects.

### 4.9 Limitations

Our study has several important limitations. The sample was restricted to a specific region of India, limiting the generalizability of the findings. The analysis did not incorporate genetic influences (69) or broader social and cultural determinants (70), both of which are known to shape the onset, progression, and treatment of mental health conditions. Furthermore, the real-world clinical setting precluded control over treatment strategies, introducing potential variability and confounding effects. Finally, although limited to a subset of cases, the use of imputation to address missing data and augment the training sample may have introduced bias and should be interpreted with caution.

Our future plan is to extend this study to multiple national and international sites to validate the identified predictive markers. Moreover, many clinicians administer multimodal treatment, that is, medicine in combination with alternative interventions such as repetitive transcranial magnetic stimulation. Our future work will also focus on understanding physiological differences across treatment modalities in predicting outcomes. Furthermore, we aim to extend this study with high-resolution data acquisition of brain and gut signals to understand cortical source dynamics underlying responses, as well as gut wave propagation in the stomach and intestine.

## Data Availability

The datasets presented in this article are not readily available due to the sensitive nature of the clinical and electrophysiological data and to protect participant privacy, in accordance with institutional ethics approval. Requests to access the datasets should be directed to pbalasub@iitk.ac.in.
