# Supplementary material for: Subtyping depression using brain–gastric electrophysiology for early prediction of antidepressant response: a multicentric clinical study
Source: Front Psychiatry. 2026 Jul 3;17:1823806. doi: 10.3389/fpsyt.2026.1823806 (PMC13377425; doi:10.3389/fpsyt.2026.1823806)
Supplement: Supplementary file 1 [file DataSheet1.pdf]

SUPPLEMENTARY MATERIALS

1) ***<u>Questionnaires question-wise differences for responders vs non-responders</u>***

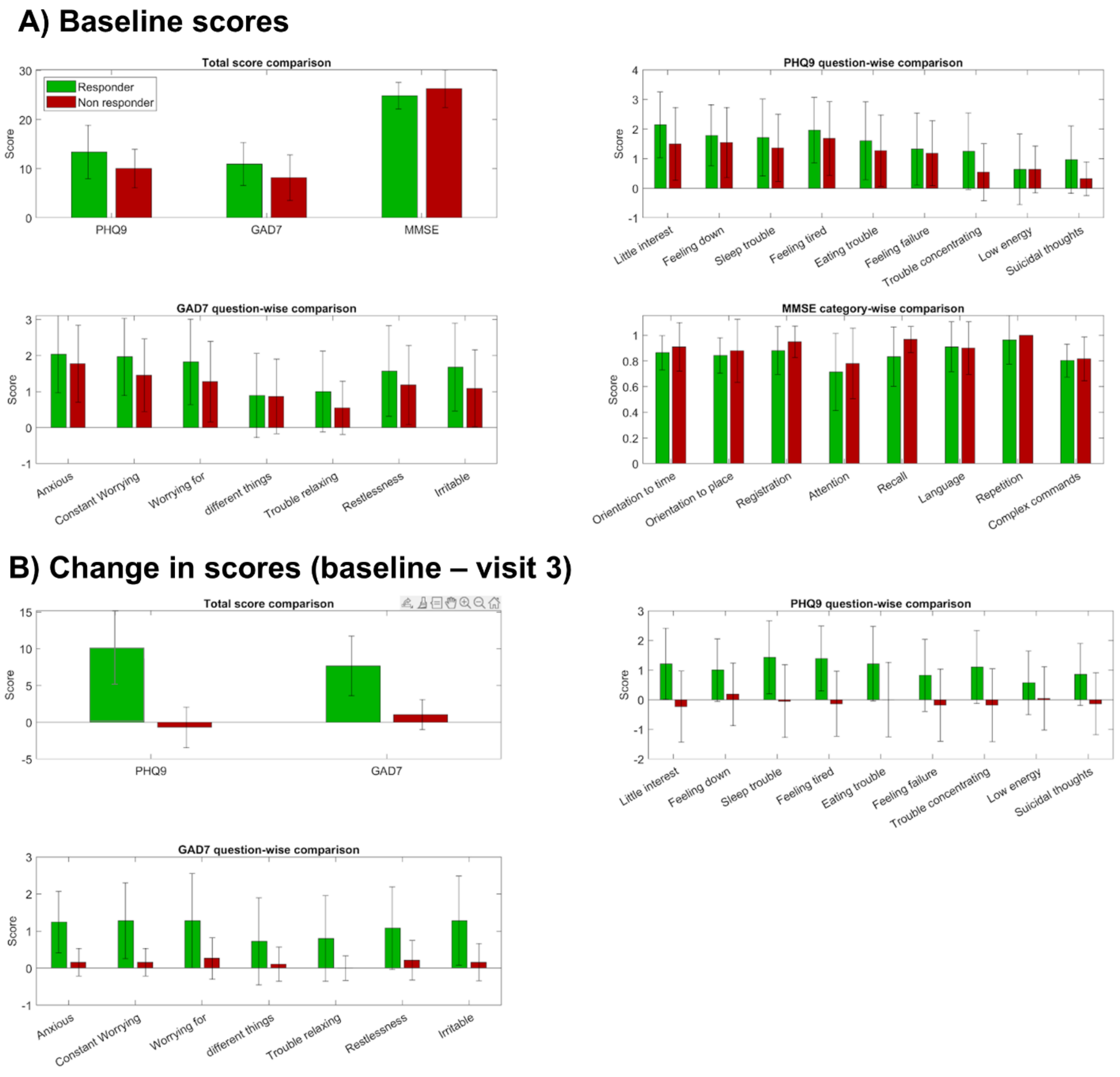

Figure I: Question-wise comparison for responders and non-responders.

From the above figure, we observe that there is no significant difference between the scores of responders and non-responders at the baseline. Post treatment, responders show significant decrease in severity across all symptoms when compared to non-responders.

Elbow and silhouette scores for baseline questionnaire-based k-means clustering

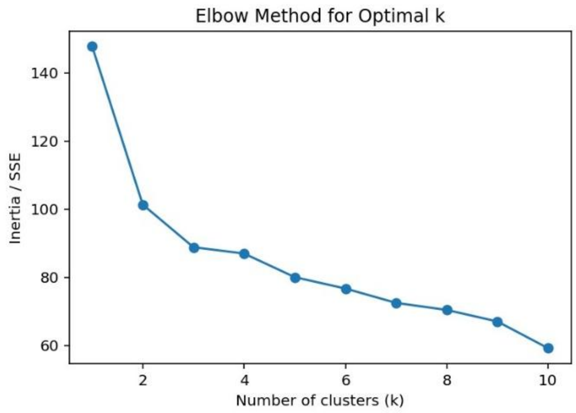

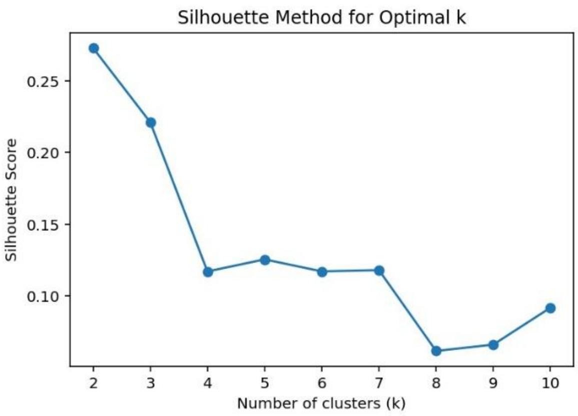

As we needed at least 3 clusters, we set the value of k to be 3 based on the above results.

2) *Feature comparison between participant groups*

BASELINE EYES OPEN TASK

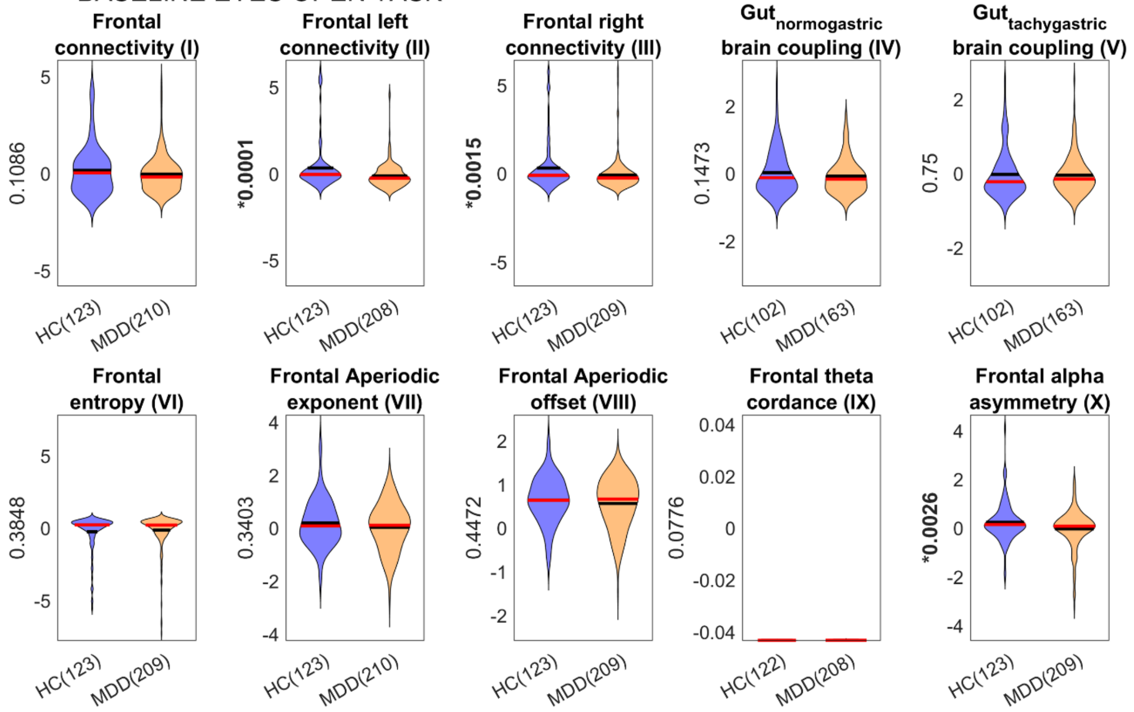

BASELINE EYES CLOSED TASK

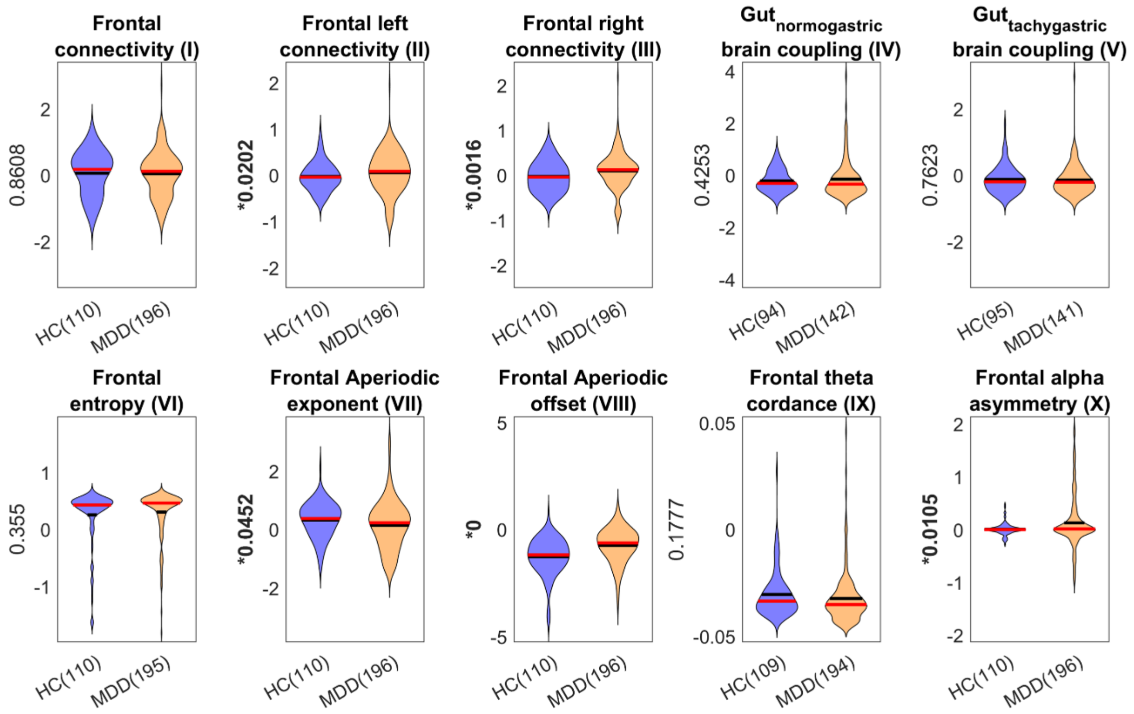

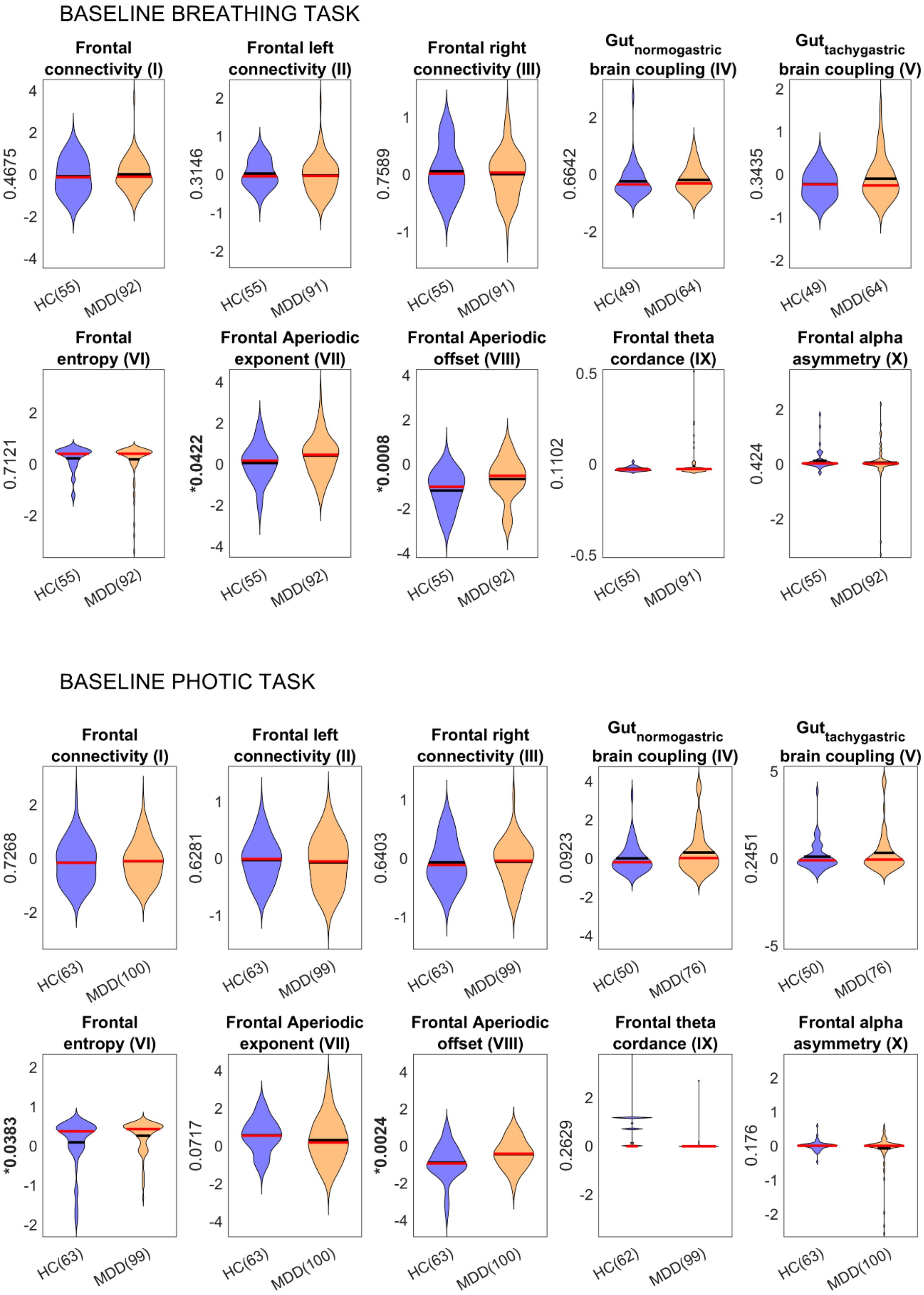

Figure II) Baseline summary feature comparison between healthy and depressed population.The p-values are uncorrected.

VISIT 2 – BASELINE EYES OPEN TASK

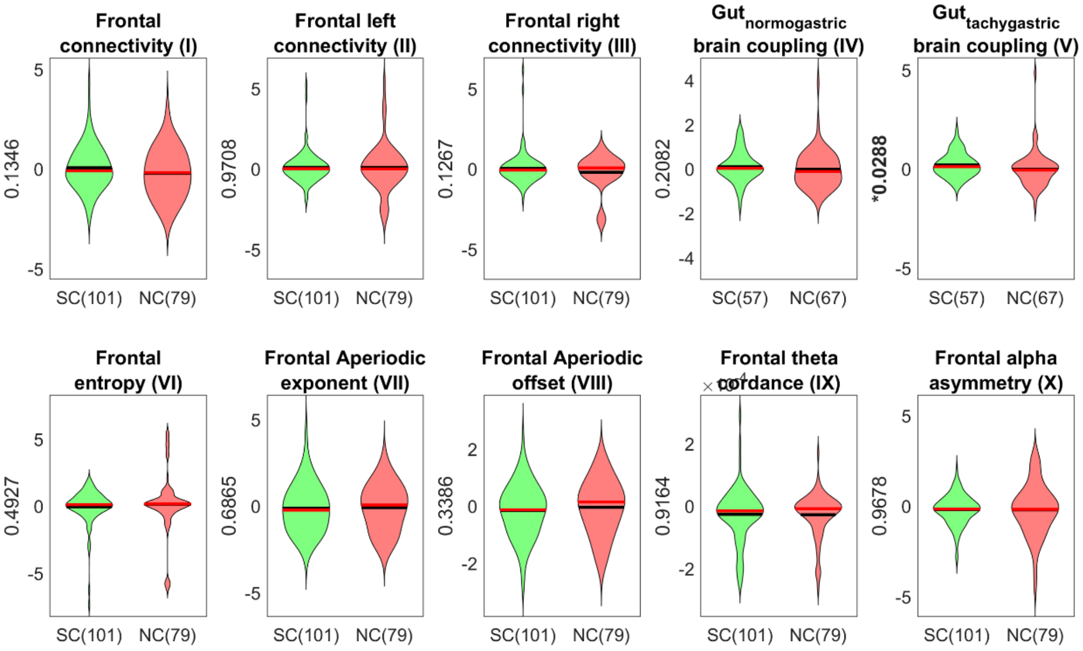

VISIT 2 - BASELINE EYES CLOSED TASK

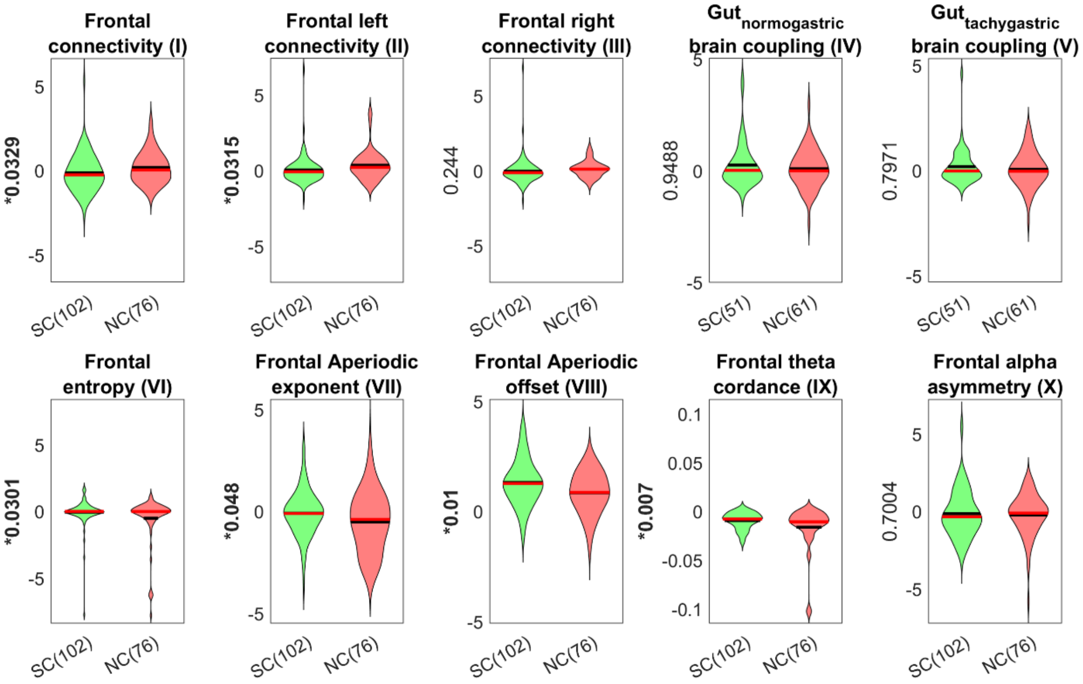

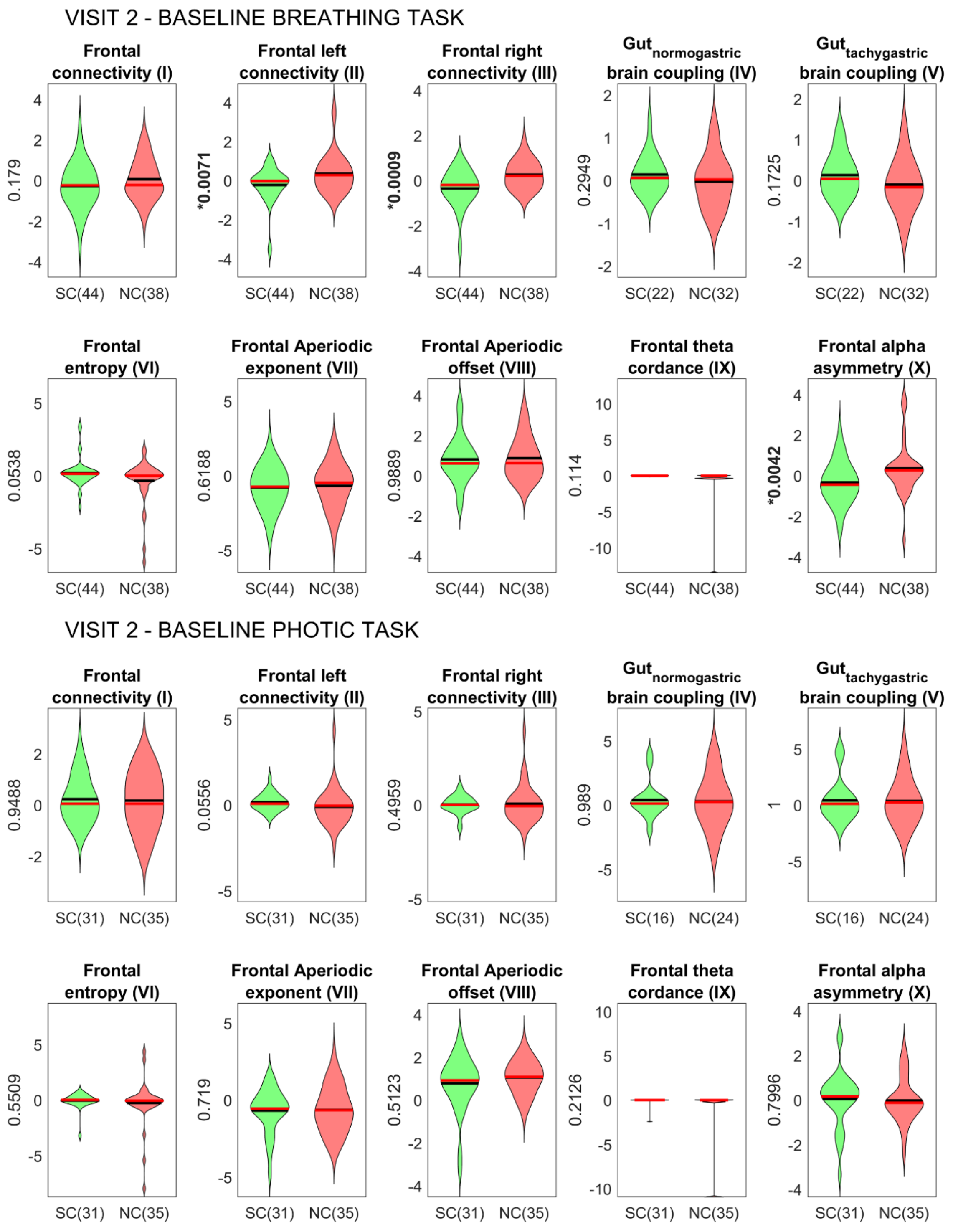

Figure III) Change in feature value at visit 2 from the baseline visit for responders and non-responders.

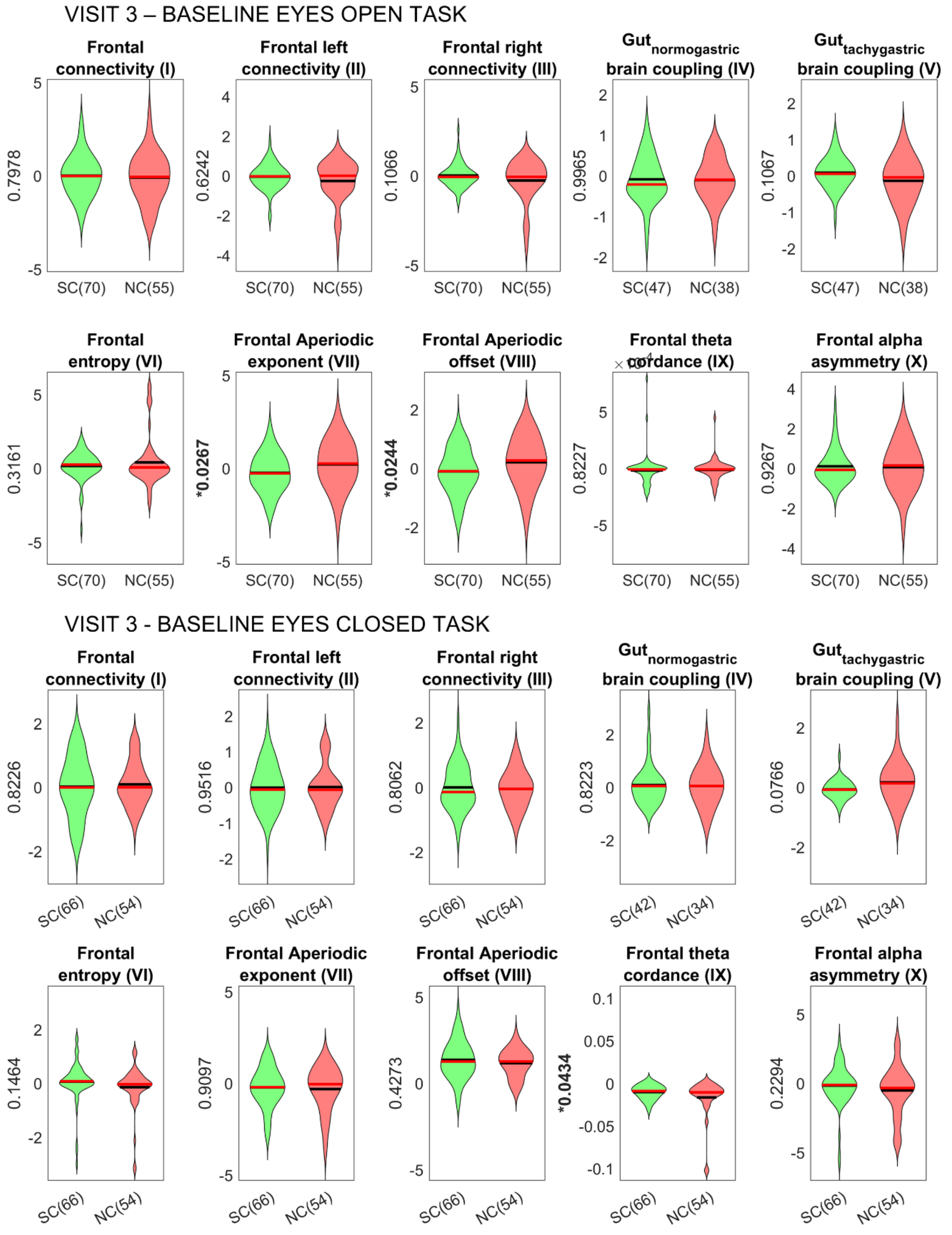
VISIT 3 – BASELINE EYES OPEN TASK
Frontal connectivity (I)
0.7978
SC(70) NC(55)
Frontal left connectivity (II)
0.6242
SC(70) NC(55)
Frontal right connectivity (III)
0.1066
SC(70) NC(55)
Gut$_{normogastric}$ brain coupling (IV)
0.9965
SC(47) NC(38)
Gut$_{tachygastric}$ brain coupling (V)
0.1067
SC(47) NC(38)
Frontal entropy (VI)
0.3161
SC(70) NC(55)
Frontal Aperiodic exponent (VII)
*0.0267
SC(70) NC(55)
Frontal Aperiodic offset (VIII)
*0.0244
SC(70) NC(55)
Frontal theta cordance (IX)
$\times 10^{-4}$
0.8227
SC(70) NC(55)
Frontal alpha asymmetry (X)
0.9267
SC(70) NC(55)
VISIT 3 - BASELINE EYES CLOSED TASK
Frontal connectivity (I)
0.8226
SC(66) NC(54)
Frontal left connectivity (II)
0.9516
SC(66) NC(54)
Frontal right connectivity (III)
0.8062
SC(66) NC(54)
Gut$_{normogastric}$ brain coupling (IV)
0.8223
SC(42) NC(34)
Gut$_{tachygastric}$ brain coupling (V)
0.0766
SC(42) NC(34)
Frontal entropy (VI)
0.1464
SC(66) NC(54)
Frontal Aperiodic exponent (VII)
0.9097
SC(66) NC(54)
Frontal Aperiodic offset (VIII)
0.4273
SC(66) NC(54)
Frontal theta cordance (IX)
*0.0434
SC(66) NC(54)
Frontal alpha asymmetry (X)
0.2294
SC(66) NC(54)

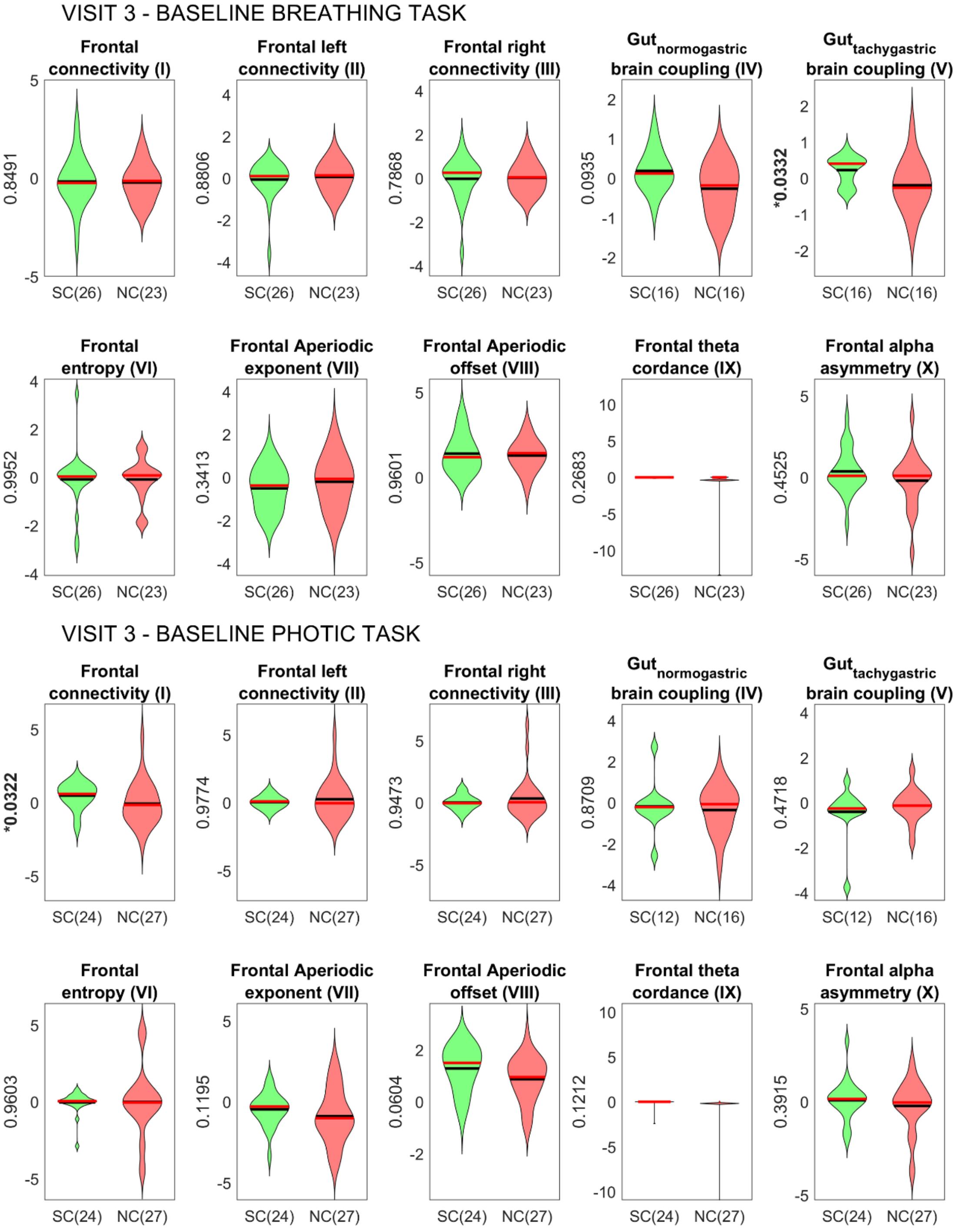

Figure IV) Change in feature value at visit 3 from the baseline visit for responders and non-responders.

From the above 3 figures (II,III,IV), we can observe that frontal connectivity with other regions, aperiodic parameters and frontal alpha asymmetry during eyes closed task are sensitive to presence of depression and also to treatment response initially. However, in the final visit, these features fail to distinguish responders and non-responders. None of the other features show reliability across more than 1 comparison for a particular task. Some of the features are distinguishing depressed population from healthy, while several other features are sensitive to treatment outcome depending on the timepoint they are probed.

3) *Final list of features, groups and their importance*

Table A: List of selected features and their groups for feature importance. T1- eyes open, T2- eyes closed, T3- breathing, T4- photic administration tasks.

GROUP	FEATURE	SHAPLEY VALUE (NC)	SHAPLEY VALUE (SC)
-	age	5.08E-05	-1.16E-05
-	gender	-0.00018	-2.60E-05
-	handedness	-0.0004	2.90E-05
-	trauma	0.001623	0.002374
-	Patient/healthy	0.019194	0.002625
Social symptoms	Phq q1 v1	0.015715	0.011284
Negative thoughts (self)	Phq q2 v1	0.000317	0.000627
Sleep	Phq q3 v1	0.000407	-0.00169
Sleep	Phq q4 v1	-0.00048	-5.70E-05
Appetite	Phq q5 v1	-6.63E-05	-0.00034
Negative thoughts (self)	Phq q6 v1	0.002472	0.003175
Social symptoms	Phq q7 v1	0.004395	0.004879
Social symptoms	Phq q8 v1	0.000426	-0.00057
Negative thoughts (self)	Phq q9 v1	0.012225	0.009761
Anxiety	Gad q1 v1	-0.00026	-0.00018

Anxiety	Gad q2 v1	0.008919	0.006164
Anxiety	Gad q3 v1	0.001075	-0.00014
Anxiety	Gad q4 v1	-0.0003	2.63E-05
Anxiety	Gad q5 v1	0.002266	0.002683
Anxiety	Gad q6 v1	0.001378	0.000807
Anxiety	Gad q7 v1	0.001451	0.000319
Cognition	mmse category 1	0.000848	0.000434
Cognition	mmse category 2	0.000145	0.000244
Cognition	mmse category 3	2.77E-05	0.00072
Cognition	mmse category 4	0.001439	-0.00032
Cognition	mmse category 5	0.000982	0.00063
Cognition	mmse category 6	-0.00037	0.000279
Cognition	mmse category 7	0	0
Cognition	mmse category 8	-3.03E-05	9.72E-05
-	Average of base Entropy Fl T1 base Entropy Fr T1 base Entropy Cl T1 base Entropy Cr T1 base Entropy Ol T1 base Entropy Or T1	-4.82E-05	0.00052
Baseline_eyes_open_global_coherence	Average of base Coh th Fl-Cl T1 base Coh th Fr-Cl T1 base Coh th Cl-Ol T1 base Coh th Cl-Or T1 base Coh alp Fl-Cl T1 base Coh alp Fr-Cl T1 base Coh alp Cl-Ol T1 base Coh bet Fl-Cl T1 base Coh bet Fr-Cl T1 base Coh bet Cl-Ol T1 base Coh bet Cl-Or T1 base Coh bet Cr-Or T1	0.001456	0.001042

Baseline_eyes_open_global_coherence	Average of base Coh th Fl-Cr T1 base Coh th Fr-Cr T1 base Coh th Cl-Cr T1 base Coh th Cr-Ol T1 base Coh th Cr-Or T1 base Coh alp Fl-Cr T1 base Coh alp Fr-Cr T1 base Coh alp Cr-Or T1 base Coh bet Fl-Cr T1 base Coh bet Fr-Cr T1 base Coh bet Cl-Cr T1 base Coh bet Cr-Ol T1	0.00058	0.00135
Baseline_eyes_open_global_coherence	Average of base Coh th Fl-Ol T1 base Coh th Fl-Or T1 base Coh th Fr-Ol T1 base Coh th Fr-Or T1 base Coh bet Fl-Ol T1 base Coh bet Fl-Or T1 base Coh bet Fr-Ol T1 base Coh bet Fr-Or T1	-0.00338	-0.00254
Baseline_eyes_open_global_coherence	Average of base Coh th Fr-Fr T1 base Coh alp Fr-Fr T1 base Coh bet Fr-Fr T1	-0.00183	-0.00125
Baseline_eyes_open_global_coherence	Average of base Coh th Ol-Or T1 base Coh alp Ol-Or T1 base Coh bet Ol-Or T1	0.008656	0.00677
Baseline_eyes_open_global_coherence	Average of base Coh alp Fl-Ol T1 base Coh alp Fr-Ol T1 base Coh alp Fr-Or T1	0.000636	5.65E-05
Baseline_Aperiodic_offset	Average of base Ap off Fl T2 base Ap off Fr T2 base Ap off Cl T2 base Ap off Cr T2 base Ap off Ol T2 base Ap off Or T2	0.014236	0.008498
Baseline_relative_power	Average of base Relative Pow th Fl T2 base Relative Pow th Fr T2 base Relative Pow th Cr T2 base Relative Pow th Fl T3 base Relative Pow th Fr T3 base Relative Pow th Cl T3 base	0.002633	5.33E-05

	Relative Pow th Cr T3 base Relative Pow th Ol T3 base Relative Pow th Or T3 base Relative Pow th Fl T4 base Relative Pow th Fr T4 base Relative Pow th Cl T4 base Relative Pow th Ol T4 base Relative Pow th Or T4		
Baseline_relative_power	Average of base Relative Pow th Cl T2 base Relative Pow th Ol T2 base Relative Pow th Cr T4	0.0043	-0.00048
Baseline_relative_power	Average of base Relative Pow alp Fl T2 base Relative Pow alp Fr T2 base Relative Pow alp Cl T2 base Relative Pow alp Cr T2 base Relative Pow alp Ol T2 base Relative Pow alp Or T2 base Relative Pow alp Fl T3 base Relative Pow alp Fr T3 base Relative Pow alp Cl T3 base Relative Pow alp Cr T3 base Relative Pow alp Ol T3 base Relative Pow alp Or T3 base Relative Pow alp Fl T4 base Relative Pow alp Fr T4 base Relative Pow alp Cl T4 base Relative Pow alp Cr T4 base Relative Pow alp Ol T4 base Relative Pow alp Or T4 base alp asymmetry occipital T4	0.002251	0.00329
Baseline_relative_power	Average of base Relative Pow bet Fl T2 base Relative Pow bet Fr T2 base Relative Pow bet Cl T2 base Relative Pow bet Cr T2 base Relative Pow bet Ol T2 base Relative Pow bet Or T2	-0.00128	-0.00085

	base Relative Pow bet Fl T3 base Relative Pow bet Fr T3 base Relative Pow bet Cl T3 base Relative Pow bet Cr T3 base Relative Pow bet Ol T3 base Relative Pow bet Or T3 base Relative Pow bet Fl T4 base Relative Pow bet Fr T4 base Relative Pow bet Cl T4 base Relative Pow bet Cr T4 base Relative Pow bet Ol T4 base Relative Pow bet Or T4		
Baseline_Aperiodic_offset	Average of base Ap off Fl T3 base Ap off Fr T3 base Ap off Cl T3 base Ap off Cr T3 base Ap off Ol T3 base Ap off Or T3	0.005292	0.002566
Baseline_breathing_theta_periodic_power	Average of base Periodic Pow th Cl T3 base Periodic Pow th Cr T3 base Periodic Pow th Ol T3 base Periodic Pow th Or T3	0.0167	0.003866
Baseline_Aperiodic_offset	Average of base Ap off Fl T4 base Ap off Fr T4 base Ap off Cl T4 base Ap off Cr T4 base Ap off Ol T4 base Ap off Or T4	0.000801	-0.00033
Baseline_absolute_power	Average of base Absolute Pow th Fl T4 base Absolute Pow th Fr T4 base Absolute Pow th Cl T4 base Absolute Pow th Cr T4 base Absolute Pow th Ol T4 base Absolute Pow th Or T4 base Absolute Pow alp Fl T4 base Absolute Pow alp Fr T4 base Absolute Pow alp Cr T4 base Absolute Pow alp Ol T4 base Absolute Pow alp Or T4 base Absolute Pow bet Fl T4 base Absolute Pow bet Fr T4 base Absolute Pow bet Cl T4	0.000671	0.000101

	base Absolute Pow bet Cr T4 base Absolute Pow bet Ol T4 base Absolute Pow bet Or T4		
Baseline_eyes_open_global_coherence	base Coh th Cr-Cr T1	0.004501	0.001367
Baseline_eyes_open_global_coherence	base Coh alp Fl-Fr T1	0.003714	0.002794
Baseline_eyes_open_global_coherence	base Coh alp Cl-Cr T1	-0.00044	-0.00048
Baseline_eyes_open_global_coherence	base Coh alp Cl-Or T1	0.016104	0.012517
Baseline_eyes_open_global_coherence	base Coh alp Cr-Ol T1	-0.00167	-0.00089
-	base Entropy Ol T2	-0.00046	0.000148
Baseline_eyes_closed_central_left_coherence	base Coh th Cl-Or T2	0.003177	-0.00067
Baseline_eyes_closed_non_central_left_coherence	base Coh bet Fl-Fl T2	0.002368	-0.00271
-	base PLI Fl-Fr T2	0.005308	0.003767
-	base PLI Ol-Ol T2	0.003825	0.005516
Baseline_breathing_low_frequency_coherence	base Coh alp Fl-Cl T3	0.014864	0.013338
Baseline_breathing_low_frequency_coherence	base Coh alp Fr-Cr T3	0.003845	0.003262
Baseline_breathing_low_frequency_coherence	base Coh alp Cr-Ol T3	-0.0002	-0.00061
Baseline_breathing_central_tachy_PAC	base tachy PAC bet top Cr T3	0.004111	0.001112
Baseline_photic_central_left_coherence	base Coh th Fr-Cl T4	0.000875	0.001967

Baseline_photic_non_central_left_coherence	base Coh alp Fr-Fr T4	-0.00098	-3.34E-05
Baseline_photic_non_central_left_coherence	base Coh alp Fr-Cr T4	0.004286	0.001904
Baseline_photic_non_central_left_coherence	base Coh alp Cr-Ol T4	0.009384	0.002897
Baseline_photic_non_central_left_coherence	base Coh bet Or-Or T4	0.003627	-0.00329
Longitudinal_Aperiodic_offset	Average of Ap exp Fl T1 Ap off Fl T1	-0.00082	-0.00091
Longitudinal_Aperiodic_offset	Average of Ap off Ol T1 Ap off Or T1	-0.00061	-0.00093
Longitudinal_eyes_open_fronto-central_left_beta_periodic_power	Average of Periodic Pow bet Fl T1 Periodic Pow bet Cl T1	0.002073	0.001369
-	Average of Periodic Pow bet Cr T1 Periodic Pow bet Ol T1 Periodic Pow bet Or T1	0.003686	0.003711
Longitudinal_fronto-central_left_absolute_power	Average of Absolute Pow th Cl T1 th cordance Cl T1	0.00011	6.06E-05
Longitudinal_eyes_open_global_coherence	Average of Coh th Cr-Ol T1 Coh bet Cr-Ol T1	0.003978	0.001764
Longitudinal_eyes_open_global_coherence	Average of Coh th Ol-Or T1 Coh alp Ol-Or T1 Coh bet Ol-Or T1	-0.0003	0.001782
-	Average of Relative Pow bet Fl T2 Relative Pow bet Fr T2 Relative Pow bet Cl T2 Relative Pow bet Cr T2 Relative Pow bet Ol T2 Relative Pow bet Or T2 Relative Pow bet Fr T3 Relative Pow bet Cl T3 Relative Pow bet	0.000907	-0.00232

	Cr T3 Relative Pow bet Ol T3 Relative Pow bet Or T3		
Longitudinal_eyes_closed_central_left_coherence	Average of Coh th Cl-Cl T2 Coh bet Cl-Cl T2	0.004102	-0.00147
Longitudinal_fronto-central_left_absolute_power	Average of Absolute Pow th Fl T3 Absolute Pow alp Fl T3 Absolute Pow alp Cl T3 Absolute Pow alp Cr T3 Absolute Pow alp Ol T3 Absolute Pow alp Or T3 Absolute Pow bet Fl T3 Absolute Pow bet Fr T3 Absolute Pow bet Cl T3 Absolute Pow bet Cr T3	0.004003	0.003358
Longitudinal_photic_non_central_left_coherence	Average of Coh th Fl-Fl T4 Coh bet Fl-Fl T4	0.006108	0.010807
Longitudinal_photic_non_central_left_coherence	Average of Coh th Fl-Cr T4 Coh th Cr-Or T4	0.005073	0.003881
Longitudinal_Aperiodic_offset	Ap exp Ol T1	0.010496	0.004676
Longitudinal_eyes_open_fronto_left_beta_relative_power	Relative Pow bet Fl T1	0.001331	-0.00235
Longitudinal_eyes_open_global_coherence	Coh bet Cr-Cr T1	0.004614	0.001228
-	th asymmetry occipital T1	-0.0014	-0.00107
Longitudinal_eyes_closed_central_left_coherence	Coh th Cl-Ol T2	0.010509	0.002536
Longitudinal_eyes_closed_non_central_left_coherence	Coh th Cr-Cr T2	0.002934	0.000754
Longitudinal_eyes_closed_non_central_left_coherence	Coh alp Fl-Fr T2	0.009293	0.006183
Longitudinal_eyes_closed_non_central_left_coherence	Coh alp Fl-Ol T2	0.002592	0.004229

Longitudinal_eyes_closed_non_central_left_coherence	Coh alp Fl-Or T2	0.001896	0.001337
Longitudinal_eyes_closed_non_central_left_coherence	Coh alp Fr-Or T2	0.016035	0.012844
Longitudinal_eyes_closed_central_left_coherence	Coh alp Cl-Cl T2	0.003974	-0.00133
Longitudinal_eyes_closed_non_central_left_coherence	Coh alp Or-Or T2	0.004425	-0.00252
Longitudinal_eyes_closed_central_left_coherence	Coh bet Cl-Ol T2	0.008804	-0.00046
Longitudinal_eyes_closed_non_central_left_coherence	Coh bet Cr-Cr T2	0.003715	-6.80E-06
Longitudinal_eyes_closed_non_central_left_coherence	Coh bet Cr-Ol T2	0.001588	-0.00275
-	PLI Fl-Or T2	0.002502	0.000891
-	PLI Fr-Fr T2	0.000541	-0.00061
Longitudinal_normo_PAC	normo PAC alp top Fl T2	0.002504	0.001151
Longitudinal_normo_PAC	normo PAC alp top Ol T2	0.000525	-0.0009
-	tachy PAC th top Fl T2	-4.27E-05	0.001114
-	tachy PAC alp top Fl T2	0.006698	0.006527
-	th asymmetry occipital T2	0.004749	0.001275
Longitudinal_low_frequency_central_asymmetry	alp asymmetry central T2	0.023277	0.019103
Longitudinal_high_frequency_frontal_asymmetry	beta asymmetry frontal T2	0.022208	0.009193
Longitudinal_breathing_fronto-central_left_theta_periodic_power	Periodic Pow th Cl T3	0.04386	0.029372

Longitudinal_breathing_fronto_left_beta_relative_power	Relative Pow bet Fl T3	0.003048	-0.00226
Longitudinal_breathing_low_frequency_coherence	Coh th Cr-Cr T3	0.000249	-0.00165
Longitudinal_breathing_low_frequency_coherence	Coh alp Fl-Cr T3	-0.00456	0.000362
Longitudinal_breathing_high_frequency_coherence	Coh bet Fr-Cr T3	0.001184	-0.00212
Longitudinal_breathing_high_frequency_coherence	Coh bet Fr-Ol T3	0.017992	0.012467
Longitudinal_normo_PAC	normo PAC brd top Or T3	0.001228	0.001127
Longitudinal_normo_PAC	normo PAC alp top Or T3	0.003027	0.006878
-	tachy PAC alp top Or T3	0.013614	0.012587
Longitudinal_breathing_central_tachy_PAC	tachy PAC bet top Cl T3	0.001239	0.000692
Longitudinal_breathing_central_tachy_PAC	tachy PAC bet top Cr T3	0.00763	0.005899
-	tachy PAC bet top Or T3	-0.00103	-0.00022
Longitudinal_high_frequency_frontal_asymmetry	beta asymmetry frontal T3	0.028463	0.011418
Longitudinal_photic_central_left_coherence	Coh th Cl-Cr T4	0.001768	2.00E-05
Longitudinal_photic_central_left_coherence	Coh alp Cl-Or T4	0.007817	0.004112
Longitudinal_photic_non_central_left_coherence	Coh alp Ol-Or T4	0.007304	0.002176
Longitudinal_photic_central_left_coherence	Coh bet Fl-Cl T4	0.00318	0.003703

Longitudinal_photic_central_left_coherence	Coh bet Fr-Cl T4	0.00918	0.007004
Longitudinal_photic_central_left_coherence	Coh bet Cl-Ol T4	0.000331	-0.00228
Longitudinal_photic_non_central_left_coherence	Coh bet Ol-Or T4	0.003007	0.001013
-	PLI Cl-Cl T4	-0.00325	2.08E-05
-	PLI Cr-Ol T4	-0.00039	0.000568
Longitudinal_low_frequency_central_asymmetry	th asymmetry central T4	0.008169	0.000334

Feature importance

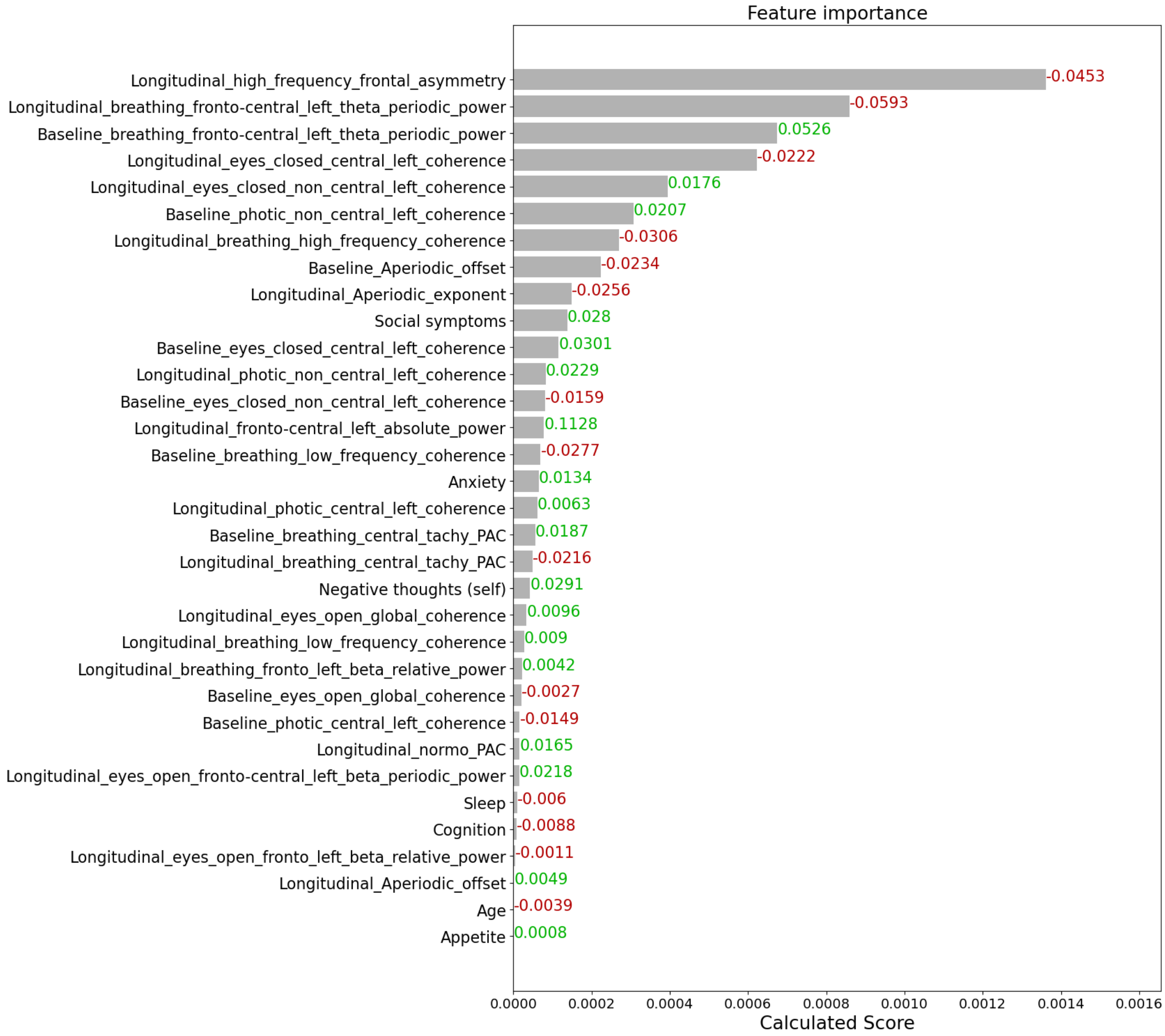

Figure V: Feature importance based on calculated score and grouping.

The slope depicts the effect of changing the feature value on shapley value. Red color depicts that increasing the feature value decreases the marginal contribution towards response and vice versa for green.

4) Symptom wise electrophysiological feature

Significant reduction in coupling of the tachygastric gut rhythm with the beta signals of central brain region, and also in overall beta coherence during breathing task, were seen in responders who had manifested higher severity than population median in appetite symptoms, negative thought about self, or social symptoms. Similarly, frontal beta asymmetry and periodic theta power in left fronto-central regions while breathing task showed significant reduction in responders with relatively higher appetite, sleep and social issues in the population. Responders with sleep issues displayed significant increase in coherence during photic administration task. Interestingly, responders with anxiety showed significant decrease in coherence in central left region, while it increased in the other regions during resting state eyes-closed task. Overall, we observed different electrophysiological biomarkers to be predictive of treatment outcome depending on their psychotype and physiological symptom presentation (**Table B**).

Table B: Significant features for specific phenotypic subtypes- Electrophysiological feature comparison for responders and non-responders in a subset of population with specific symptom severity, with relatively higher symptom score than the population median. The above table highlights the feature group from the top 20 that are significant after Bonferonni correction for multiple comparisons, their corrected p-value, the mean feature value and N of SC and NC groups exhibiting significant change and no change in mental health for the specific subset of cohort.

Symptom group	Electrophysiology feature	mean_NC	mean_SC	corrected p_value
Appetite	Baseline breathing left fronto-central theta periodic power	-0.160 (± 0.432)	0.700 (± 1.146)	0.034
	Baseline photic left non central coherence	-0.542 (± 0.294)	-0.104 (± 0.365)	<0.001
	Longitudinal breathing central tachy PAC	0.282 (± 0.596)	-0.176 (± 0.379)	0.042
	Longitudinal breathing beta coherence	0.192 (± 0.115)	-0.278 (± 0.328)	<0.001
	Longitudinal beta frontal asymmetry	0.259 (± 0.712)	-0.612 (± 1.121)	0.033
	Longitudinal breathing left fronto-central theta periodic power	0.235 (± 0.715)	-0.969 (± 0.823)	<0.001
Anxiety	Longitudinal eyes closed left non central coherence	-0.323 (± 0.330)	0.377 (± 0.776)	<0.001
	Longitudinal eyes closed left central coherence	0.261 (± 0.291)	0.063 (± 1.238)	0.041
Negative thoughts self	Longitudinal breathing central tachy PAC	0.356 (± 0.611)	-0.143 (± 0.395)	0.020
	Longitudinal eyes closed left central coherence	1.154 (± 1.946)	0.032 (± 1.259)	0.031
	Longitudinal breathing beta coherence	0.772 (± 0.793)	-0.054 (± 0.469)	<0.001

Sleep	Longitudinal photic non central beta coherence	-0.196 (± 0.461)	0.222 (± 0.449)	0.003
	Longitudinal photic central beta coherence	-0.070 (± 0.299)	0.248 (± 0.525)	0.040
	Longitudinal beta frontal asymmetry	0.105 (± 0.833)	-0.588 (± 1.037)	0.006
	Longitudinal breathing left fronto-central theta periodic power	0.078 (± 0.753)	-0.825 (± 0.748)	<0.001
Social symptoms	Longitudinal breathing central tachy PAC	0.291 (± 0.565)	-0.130 (± 0.390)	0.039
	Longitudinal eyes closed left central coherence	0.723 (± 1.763)	-0.106 (± 1.150)	0.024
	Longitudinal breathing beta frequency coherence	0.501 (± 0.559)	-0.039 (± 0.414)	<0.001
	Longitudinal beta frontal asymmetry	0.315 (± 0.512)	-0.480 (± 0.790)	<0.001
	Longitudinal breathing left fronto central theta periodic power	0.370 (± 0.910)	-0.753 (± 0.505)	<0.001

5) *Iterative Imputation efficacy testing*

The details of the subjective missing data used for imputation is provided in Table C. The efficiency of imputation was computed through various models, and the best model was selected (Iterative imputer, Table D).

Table C: Details of subjects and their missing data.

Subject ID	Output label	Task and visit missing
150	SC	Breathing task – visit 2
169	HC	Photic task – visit 2
204	HC	Breathing and photic tasks – visit 1
223	SC	Breathing task – visit 1
229	HC	Photic task – visit 1
313	SC	Photic task – visit 2
316	NC	Photic task – visit 2

Next, about two imputation techniques were compared with null distribution imputation by randomly dropping 20% of the values and imputing them. The randomization was done for 10 iterations and the mean and std dev for each metric has been reported in Table E. Iterative imputer out-performed median imputation by a huge margin.

Table D: Comparison between various imputation techniques

Imputation technique	R2 coefficient	Pearson correlation	Mean squared error
Iterative imputer (null)	-0.6411 ± 0.0967	0.1169 ± 0.0160	1.7806 ± 0.0632
Median imputer	0.0766 ± 0.0104	0.2960 ± 0.0138	1.0035 ± 0.0374
Iterative imputer	0.5434 ± 0.0238	0.7659 ± 0.0108	0.4956 ± 0.0192

In order to validate the efficiency of data imputation and study its effect on model performance, we trained and tested the prediction model with and without imputed data following Synthetic Minority OverSampling Technique (SMOTE), and compared the model performance. A 5-fold cross validation strategy was performed.

Table E: Model performance comparison for imputed vs raw data

Model class	Data nature	Mean accuracy (%)
3 class model	Imputed (N subjects = 48)	77 ± 2
	Raw (N = 40)	74 ± 4
2 class model	Imputed (N subjects = 28)	79 ± 3
	Raw (N = 24)	72 ± 3
